# Upregulation of miR-146a-5p and miR-146b-5p limits IL-1β-mediated signaling in adipose tissue during polytrauma by downregulating IRAK1

**DOI:** 10.3389/fimmu.2026.1658504

**Published:** 2026-03-04

**Authors:** Antonia Mortsch, Julian Roos, Rebecca Halbgebauer, Ludmila Lupu, Annette Palmer, Anja Werberger, Ulrich Stifel, Martin Wabitsch, Markus Huber-Lang, Julia Zinngrebe, Pamela Fischer-Posovszky

**Affiliations:** 1 Department of Pediatrics and Adolescent Medicine, Ulm University Medical Centre, Ulm, Germany; 2 Institute of Clinical and Experimental Trauma Immunology, Ulm University Medical Centre, Ulm, Germany; 3 Department of Pediatrics and Adolescent Medicine, Division of Pediatric Endocrinology and Diabetes, Ulm University Medical Centre, Ulm, Germany; 4 German Center for Child and Adolescent Health (DZKJ), partner site Ulm, Ulm, Germany

**Keywords:** adipocytes, adipose tissue, interleukin-1, miRNAs, polytrauma

## Abstract

MicroRNAs (miRNAs) are small non-coding RNAs and play a crucial role in the regulation of inflammation. White adipose tissue (WAT) covers the body and internal organs in subcutaneous and visceral fat depots, respectively, and represents an important source of circulating miRNAs. The role of WAT and its miRNAs in the context of polytrauma is incompletely understood. However, evidence is accumulating that WAT contributes to the severe inflammatory response observed in polytrauma patients. Therefore, we analyzed the miRNA expression in inguinal WAT depots in a standardized mouse model of polytrauma and hemorrhagic shock (PT+HS). Here, we identified miR-146a-5p and miR-146b-5p to be upregulated upon PT+HS. In an *in-vitro* model of human white adipocytes, we found miR-146a-5p to be upregulated by IL-1β-induced NF-κB activation. Both, miR-146a-5p and miR-146b-5p, in turn, dampened IL-1β-induced inflammation in human adipocytes. Using target gene prediction tools, we further confirmed IRAK1 as target of miR-146a-5p, and potentially also miR-146b-5p, underlining the importance of IRAK1 in IL-1β-induced proinflammatory signaling. Thus, miR-146a-5p and miR-146b-5p act as suppressors of IL-1β-induced proinflammatory signaling in human adipocytes during trauma, and blockage of IL-1β or mimics of miR-146a-5p and miR-146b-5p might represent a potential future therapeutic avenue for severe traumatic and inflammatory conditions.

## Introduction

MicroRNAs (miRNAs) are small non-coding RNAs, only 18–25 nucleotides in length, that play an important role in the post-transcriptional regulation of gene expression (14). They have been implicated in the regulation of various cellular processes, such as cell growth, differentiation, development, and apoptosis (52). Multiple studies have highlighted that miRNAs, besides intracellular expression, are also present in body fluids such as serum, plasma, saliva, urine, and breast milk, and, thus, regulate protein expression through auto-, para-, and endocrine mechanisms (60, 70). miRNAs are generated in the nucleus and exported as pre-miRNAs into the cytosol where they are processed by the ribonuclease Dicer into mature miRNAs (53).

Studies using adipocyte-specific Dicer-knockout mice showed that white adipose tissue (WAT) is a main source of circulating miRNAs (60). WAT has long been regarded solely as energy reservoir, however, research of the last decades identified WAT as important endocrine organ regulating vital processes such as homeostasis, hemostasis, and inflammation (26) via its secretion products, called adipokines (49). Evidence is accumulating that WAT contributes to the inflammatory response observed in patients with polytrauma (21, 48), a leading cause of death among people under the age of 45 years (72).

Patients with polytrauma are seriously injured and suffer from a massive systemic inflammatory response syndrome (SIRS) leading to organ dysfunction (24). Interestingly, changes in the miRNA expression profile were observed within the cerebral cortex after brain injury, after acute burn injury or after trauma-induced hemorrhagic shock (16, 29, 34). Intensive investigations are carried out in the field of trauma research to enable early identification of patients who are at increased risk of multiple organ failure, and to reduce systemic inflammation and its detrimental sequelae following polytrauma (22).

Given the intricate intertwining of adipose tissue, miRNAs, and trauma, we hypothesized that the miRNA expression profile of adipose tissue is altered during polytrauma, and that adipose tissue-derived miRNAs modulate the trauma-induced immune response in an auto- and paracrine manner. For this purpose, we used a murine model of polytrauma with hemorrhagic shock (PT + HS) and subsequently performed a miRNA array analysis of inguinal WAT (iWAT) depots. Various miRNAs, amongst them miR-146a-5p and miR-146b-5p, were differentially regulated upon PT + HS in murine iWAT depots. Using an *in-vitro* model of adipocytes, we identified miR-146a-5p and miR-146b-5p to ameliorate IL-1β-induced pro-inflammatory signaling in human adipocytes. On the molecular level miR-146a-5p, and potentially also miR-146b-5p, causes downregulation of IRAK1, a kinase important for IL-1β-mediated signal transduction.

## Materials and methods

### Animal experiments

The study is reported in accordance with ARRIVE guidelines. The animal experiments were performed according to the National Institutes of Health guidelines for the use of laboratory animals and were approved by the federal authorities for animal research, Tübingen, Germany. Male C57BL/6 mice aged 10–12 weeks with a mean body weight of 29.5 g, kindly provided by T. E. Mollnes (University of Oslo), were randomly assigned to sham treatment or PT + HS (n=5 animals per group) (9, 66). No specific inclusion/exclusion criteria or outcome measures were deemed relevant for this specific study. In brief, mice were anesthetized with 2.5% sevoflurane (Abbott, Wiesbaden, Germany) in oxygen throughout the experiment before they were subjected to thoracic trauma, closed head injury, and femur fracture including soft tissue injury as described previously (63). Pressure-controlled hemorrhage was induced and monitored by a microcatheter in the femoral artery. A mean arterial pressure of around 30 ± 5 mmHg was maintained for 60 minutes. After resuscitation of the animals with a balanced electrolyte solution, inguinal WAT depots were removed 4 hours after trauma. Euthanasia was performed by thoracic opening under deep narcosis and terminal blood sampling from the right ventricle. Due to the nature of the animal experiment, investigators were not blinded during the animal study protocol.

### Cell culture

Simpson-Golabi-Behmel syndrome (SGBS) (15, 61) preadipocytes were cultured as described (12, 61). Adipogenic differentiation was induced three days after seeding by washing the cells once with DPBS (Gibco) before adding serum-free DMEM-F12 (Gibco) supplemented with 100 U/ml penicillin/streptomycin, 17 µM pantothenate, 33 µM biotin, 0.01 mg/ml transferrin, 20 nM insulin, 100 nM cortisol, 0.2 nM T3, 25 nM dexamethasone, 250 μM IBMX and 2 μM rosiglitazone. After 4 days, medium was changed and serum-free DMEM-F12 supplemented with 100 U/ml penicillin/streptomycin, 17 µM pantothenate, 33 µM biotin, 0.01 mg/ml transferrin, 20 nM insulin, 100nM cortisol, and 0.2 nM T3 was added. Cells were used on different days of adipogenic differentiation for further experiments.

HEK293 cells are available from ATCC and were used for the Dual Luciferase Reporter Assay. They were cultured in DMEM (Gibco) supplemented with 10% FCS, 100 U/ml penicillin/streptomycin, 1% L-Glutamine, 1% sodium pyruvate and 1% MEM NEAA. Two days after thawing, the cells were subcultured and allocated to two 175 cm² flasks. After 2–3 days of expansion, cells were seeded in 48-well-plate dishes at a seeding density of 80,000 cells/well for the experiments.

We used an *in-vitro* model mimicking a polytrauma micromilieu as recently established in multipotent mesenchymal stromal cells (22). SGBS adipocytes were stimulated with trauma-relevant concentration of IL-1β (200 pg/ml), IL-6 (500 pg/ml), IL-8 (50 pg/ml), C3a (500 ng/ml), and C5a (10 ng/ml) (4, 5, 18, 22, 23, 71). In brief, on day 14 or 15 of adipogenic differentiation, medium was changed to serum-free DMEM-F12 supplemented with 100 U/ml penicillin/streptomycin, 17 µM pantothenate, 33 µM biotin, 0.01 mg/ml transferrin, 20 nM insulin, 100 nM cortisol, 0.2 nM T3 and the above-named factors (either as a mix or individually), or DPBS + 0.1% BSA as vehicle control. 4 hours after stimulation, supernatants were collected and RNA isolated from the cells. Recombinant human IL-1β, IL-6, IL-8, C5a or C3a were obtained from PeproTech (Cranbury, New Jersey, USA) or Bio-Techne (Minneapolis, Minnesota, USA), respectively. Disulfiram as inhibitor of the NF-κB signaling pathway was obtained from Merck (Darmstadt, Germany). The MEK/ERK inhibitor trametinib was obtained from Selleck Chemicals (Houston, Texas, USA).

### Cell viability assay

Cell viability was measured using CellTiter-Glo^®^ assay (G7571, Promega) according to the manufacturer’s manual.

### Affymetrix microRNA array

200 ng of total RNA were labelled using the FlashTag™ Biotin HSR RNA Labeling Kit (Genisphere, Hatfield, PA, USA). miRNAs were hybridized to Affymetrix™ miRNA 4.0 arrays. Then, arrays were stained and washed on a GeneChip Fluidics Station 450 (Affymetrix). The arrays were analyzed by the Affymetrix GeneChip Scanner 3000 and the Affymetrix Expression Console™ software. As only mouse miRNAs were used, the raw feature data were normalized using the RMA + DBAG algorithm and log2 intensity expression. A miRNA-transcriptome analysis was performed using BRB-ArrayTools developed by Dr. Richard Simon and BRB-ArrayTools Development Team (http://linus.nci.nih.gov/BRB-ArrayTools.html) (47). Probesets present in at least 40% of the samples were used, and miRNAs differentially regulated among the two groups were identified by using an unpaired, two-tailed student’s t-test and were considered statistically significant if their p-value was ≤ 0.05.

### Target gene prediction

miRNA target genes were predicted using TargetScan 8.0 (https://www.targetscan.org/vert_80/) (35), miRWalk 3.0 (http://mirwalk.umm.uni-heidelberg.de/) (55) and WikiPathway (37). The intersection of the predicted miRNA targets was further analyzed with genes of a signaling pathway predicted by WikiPathways.

### Transfection of miRNAs and knockdown experiments

On day 7 of adipogenesis, SGBS adipocytes were transfected with 50 nM miRIDIAN^®^ microRNA human hsa-miR-146a-5p-Mimic (C-300630-03-0005, Dharmacon, Lafayette, Colorado, USA), 50 nM miRIDIAN^®^ microRNA human hsa-miR-146b-5p-Mimic (C-300754-03-0005, Dharmacon) (see Appendix) or miRIDIAN^®^ microRNA Mimic Negative Control #1 (CN-001000-01-20, Dharmacon) using 0.66 μl/cm² Lipofectamine 2000 (Invitrogen, Waltham, Massachusetts, USA) according to the manufacturer’s protocol. Transfection efficiency was validated after seven days by analyzing the levels of the transfected miRNA mimic by qRT-PCR.

For IRAK1, TRAF6, and REL knockdown studies 20 nM On-TARGETplus human IRAK1 (3654) siRNA-SMARTpool (L-004760-00-0005), On-TARGETplus human TRAF6 (7189) siRNA-SMARTpool (L-004712-00-0005), On-TARGETplus human REL (5966) siRNA-SMARTpool (L-004768-00-0005) and On Target Plus Non-targeting siRNA control #1 (D-001810-01-05, Dharmacon) were used. Transfection was performed with 0.66μl/cm² Lipofectamine RNAiMAX (Invitrogen) on day 11 of adipogenesis and validated after 72 hours by analyzing the knockdown efficiency of the target gene by qPCR.

### Dual Luciferase Reporter Assays

pmirGLO Dual Luciferase miRNA target expression vector (Promega) was used for assessing interaction of miR-146a-5p with a predicted binding site in the REL mRNAs. The potential REL-binding site of miR-146a-5p was annotated by TargetScan 8.0 at position 8912–8919 of the REL 3’ UTR (35, 38). The cDNA sequence for REL was obtained from Ensembl (19, 73). SnapGene version 4.2.11 was used for cloning primer design: fwd 5’-GAT CGA GCT CAT CCC AGC AGA ATA CCA AA-3’; rev 5’-GAT CTC TAG AGC ATT TTG GCA TTT TAA AAA CAA CT-3’ (33). The binding site was cloned into the 3′UTR of the firefly luciferase reporter gene encoded on pmirGLO. For dual luciferase reporter assays, 25 ng of dual luciferase vector containing the predicted binding sites of miR-146a-5p or 25 ng of the pmirGLO-vector as empty vector and 100 nM of miR-146a-5p mimic or mimic control were co-transfected into HEK293 cells using Lipofectamine 2000 (Invitrogen, Waltham, Massachusetts, USA) for 24 hours. The Dual-Glo Luciferase Assay System (Promega) was used for quantifying luciferase activity in a microplate reader (Tecan).

### RNA isolation

RNA was isolated using Direct-zol RNA Miniprep Kit (Zymo Research) according to the manufacturer’s manual. For mRNA quantification, cDNA was synthesized using SuperScript II Reverse Transcriptase (Thermo Fisher, Waltham, Massachusetts, USA) with random primers (Thermo Fisher). RT–qPCR was performed using Sso Advanced Universal Probes Supermix (Bio-Rad, Hercules, California, USA) or iTaq Universal SYBR Green Supermix (Bio-Rad) on a Bio-Rad CFX Connect Real-Time PCR Detection System with the following protocol: 95 °C for 30 s, then 40 cycles of 95 °C for 5 s followed by 60 °C for 30 s. For miRNA quantification, total RNA was reverse-transcribed with the miRCURY LNA RT Kit (Qiagen, Venlo, Netherlands) and analyzed by the miRCURY LNA SYBR Green PCR Kit. qRT–PCR for miRNAs was also performed on a Bio-Rad CFX Connect Real-Time PCR Detection System with the following protocol: 42 °C for 60 minutes, 95 °C for 5 minutes, stored at 4 °C. Results were normalized to sno44 (=human, miRNA), sno68 (=mouse, miRNA) and *HPRT* (mRNA) using the ΔCt-method (32). Primer sequences can be found in **Supplementary Table S1**. For miRNA PCR primers see **Supplementary Table S2**.

### Western blot

Cells were washed with PBS and lysed in lysis buffer [10 mM Tris-HCl, 150 mM NaCl, 2 mM EDTA, 1% Triton X-100, 10% glycerol, 1 mM DTT, cOmplete Protease Inhibitor Cocktail (Roche, Basel, Switzerland)], with or without PhosStop (Roche, Basel, Switzerland). Lysates containing 13-15 µg protein were separated by electrophoresis using Bolt 4-12% Bis-Tris Plus gel (Thermo Fisher) and 1X Bolt MES SDS running buffer (Thermo Fisher). Achieving protein transfer onto nitrocellulose membranes, the BioRad Trans-Blot Turbo Transfer System was used according to the manufacturer’s manual. The membranes were blocked in blocking buffer (5% milk in TBST) for one hour at room temperature. Then, membranes were incubated with primary antibodies followed by incubations with the appropriate secondary antibodies. Protein expression was analyzed on a ChemiDoc MP Imager (Bio-Rad) with ImageLab software (Bio-Rad).

### Antibodies used for western blots

Adiponectin (GTX112777, polyclonal, rabbit, GeneTex, dilution 1:1,000), c-REL (82000, monoclonal, rabbit, Cell Signaling Technology (CST), dilution 1:1,000), IRAK1 (SC-5287, monoclonal, mouse, Santa Cruz Biotechnology, dilution 1:200), pAKT (S473) (9271, polyclonal, rabbit, CST, dilution 1:1,000), AKT (9272, polyclonal, rabbit, CST, dilution 1:1,000), pERK 1/2 (Thr202/Tyr204) (9106, monoclonal, mouse, CST, dilution 1:2,000), ERK 1/2 (M5670, polyclonal, rabbit, Sigma-Aldrich, dilution 1:10,000), GLUT4 (PA1722, polyclonal, rabbit, Boster, dilution 1:1,000), pIκBα (Ser32/36) (9246, monoclonal, mouse, CST, dilution 1:1,000), IκBα (Ser32/36) (9242, polyclonal, rabbit, CST, dilution 1:1,000), Leptin (RD181001220, polyclonal, rabbit, bioVendor, dilution 1:1,000), PLIN1 (Ab3526, polyclonal, rabbit, Abcam, dilution 1:1,000), PPARγ (2443, monoclonal, rabbit, CST, dilution 1:1,000), TRAF6 (PA5-29622, polyclonal, rabbit, Invitrogen, dilution 1:1,000) and GAPDH (12004168, Rhodamine, Bio-Rad, dilution 1:5,000) were used as primary antibodies. Goat Anti-Mouse IgG (12005867, polyclonal, Star Bright Blue 520, Bio-Rad, dilution 1:5,000), Goat Anti-Mouse IgG (12004159, polyclonal, Star Bright Blue 700, Bio-Rad, dilution 1:5,000), and Goat Anti-Rabbit IgG (12004162, polyclonal, Star Bright Blue 700, Bio-Rad, dilution 1:5,000) were used as secondary antibodies.

### ELISA

Cell culture supernatants were collected and stored at -20 °C until further analysis. The human IL-6 uncoated ELISA-Kit, the human IL-8 uncoated ELISA-Kit, and the human CCL2 (MCP-1) uncoated ELISA-Kit (all from Invitrogen) were used to determine concentrations of IL-6, IL-8, and CCL2 (MCP-1) according to the manufacturer’s instructions. For values exceeding the detection range of the ELISA assay, the highest value of the standard curve was used.

### Statistical analysis

Data were analyzed using GraphPad Prism software (version 9.3.1) (LLC., San Diego, California, USA). For comparison of two groups, a t-test was used. For comparison of more than two groups, one-way ANOVA (for one independent variable), two-way ANOVA (for two independent variables) or mixed-effects analysis were used. Multiple comparisons were corrected by Tukey test (comparing every mean with every other mean), Dunnett´s test (comparing every mean to control) or the Šídák correction test (assuming that each comparison is independent of another comparison). All experiments were performed at least three times. Normal (Gaussian) distribution of data was assumed and p-values ≤ 0.05 were considered statistically significant. All data are presented as mean ± SEM.

### Data availability

All data generated or analysed during this study are included in this article or have been deposited on the NCBI GEO website (accession number: GSE302289).

## Results

### MiR-146a-5p and miR-146b-5p are upregulated in inguinal WAT depots in a murine model of polytrauma *in vivo*

**Figure 1:**
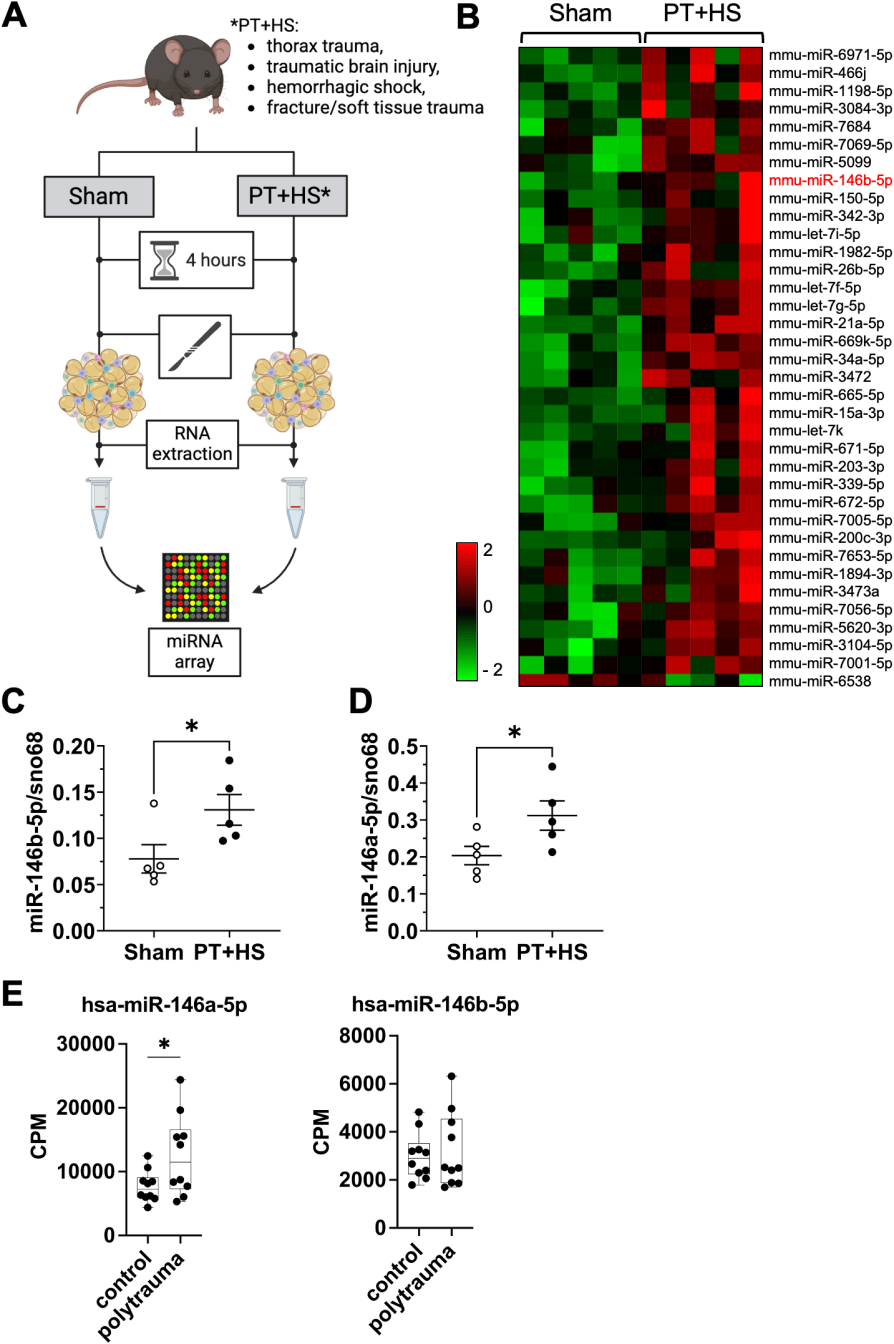
MiR-146a-5p and miR-146b-5p are upregulated in inguinal WAT depots in a murine model of polytrauma *in vivo*. **(A, B)** The microRNA (miRNA) expression profile of inguinal white adipose tissue (iWAT) from five sham-treated mice and five mice with polytrauma and hemorrhagic shock (PT+HS) was assessed 4 h after treatment by an Affymetrix miRNA microarray. Depicted are differentially regulated miRNAs between sham and PT+HS with a p-value ≤ 0.05 (Graphic in panel A was created with BioRender.com). **(C, D)** miR-146b-5p **(C)** and miR-146a-5p **(D)** expression in iWAT of sham and PT+HS mice validated by RT-qPCR. **(E)** Human plasma miR-146a/b-5p was assessed in publicly available miRNA sequencing data [Gene Expression Omnibus (GEO): GSE223151 (56)]. Multimapping-adjusted miRNA read count tables provided by the original study were downloaded and processed in R. Raw miRNA counts were normalized to counts per million (CPM) to account for differences in sequencing depth across samples. For hypothesis-driven analysis of individual miRNAs, group differences between control and trauma samples were assessed using Welch’s t-test on CPM-normalized values. Statistics: unpaired, two-tailed student’s t-test, p-values ≤ 0.05 were considered statistically significant **(B)**, unpaired two-tailed t-test, *p < 0.05 **(C, D)**.

We recently showed in a mouse model of polytrauma with thoracic trauma, traumatic brain injury, and femur fracture including soft tissue injury and hemorrhagic shock (PT+HS) that already four hours after injury inflammation and cell death are detectable in inguinal WAT (iWAT) depots not directly hit by the traumatic force vector itself (48). To identify WAT-derived miRNAs regulated in the context of trauma, we therefore performed a miRNA array on iWAT depots of mice 4 hours after PT+HS versus sham treatment (**Figure 1A**), and discovered 36 differentially regulated miRNAs (**Figure 1B**). Adipose identity of the tissue used to analyze the miRNA expression has been confirmed previously by histological analysis and expression of adipogenic marker genes (48). miRNAs evolutionarily conserved between mouse and human with a fold change (FC) ≥ 2 for PT+HS versus sham (**Supplementary Table 3**) were validated by qRT-PCR (**Figures 1C**; **Supplementary Figure 1**). Here, miR-146b-5p was significantly upregulated in iWAT depots upon PT+HS compared to sham (**Figures 1C**; **Supplementary Figure 1**). Notably, miR-146b-5p and miR-146a-5p are members of the same miRNA family, sharing similar properties (42). In line with this, we observed that miR-146a-5p was also upregulated upon PT+HS (**Figure 1D**; miRNA array: p = 0.066, FC = 3.44). To assess human relevance of miR-146a/b-5p upregulation during polytrauma, we evaluated the expression of miR-146a/b-5p in plasma from healthy controls and trauma patients in publicly available miRNA sequencing data (GSE223151) (56). Here, miR-146a-5p, but not miR-146b-5p, was significantly upregulated in patients with polytrauma (**Figure 1E**).

### MiR-146a-5p and miR-146b-5p are upregulated in a model system of traumatized human white adipocytes

**Figure 2:**
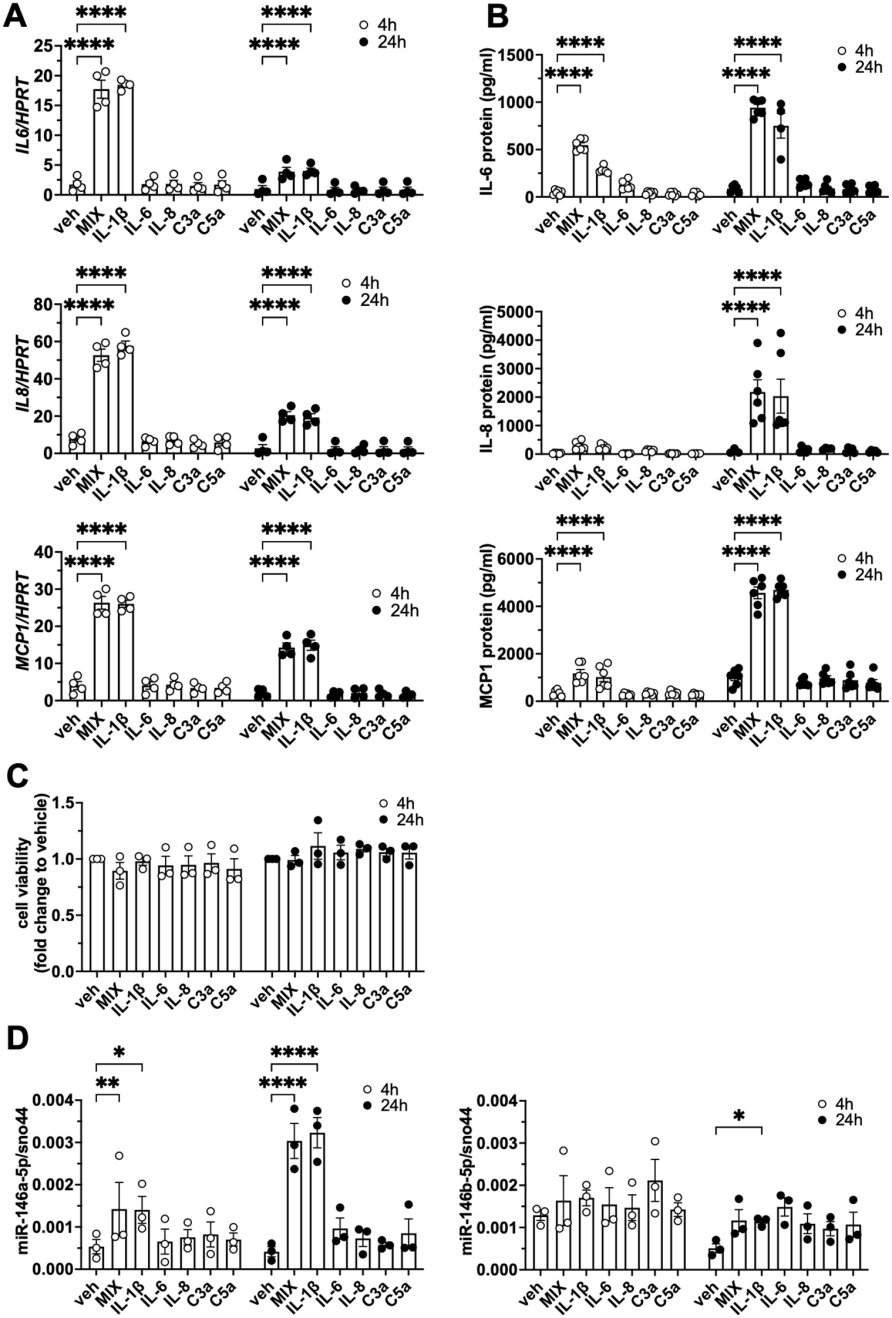
MiR-146a-5p and miR-146b-5p are upregulated in a model system of human white adipocytes. SGBS adipocytes were treated with different trauma-relevant factors, i.e. IL-1β, IL-6, IL-8, C3a, C5a, a mix thereof or left untreated (vehicle control). Total RNA was isolated and media supernatant was harvested 4 h and 24 h post-stimulation. **(A)***IL6*, *IL8* and *MCP1* mRNA expression were assessed by RT-qPCR with *HPRT* as reference gene. The results are displayed as mean ± SEM of four independent experiments performed in triplicates. **(B)** IL-6, IL-8, and MCP1 were measured in media supernatants by ELISA. The results are displayed as mean ± SEM of three independent experiments performed in duplicates. **(C)** Cell viability was measured by CellTiterGlo (CTG) assay. **(D)** miR-146a-5p and miR-146b-5p expression was assessed by RT-qPCR with sno44 as reference gene. The results are displayed as mean ± SEM of three independent experiments performed in triplicates. Statistics: two-way ANOVA or mixed effects analysis with Dunnett correction, *p < 0.05, **p < 0.01, ****p < 0.0001.

Based on (22), we set up an *in-vitro* model mimicking polytrauma in human adipocytes. The human Simpson-Golabi-Behmel Syndrome (SGBS) cells are a well-characterized model system of human preadipocytes which can effectively be differentiated into adipocytes by using an adipogenic cocktail (**Supplementary Figure 2**). During the course of adipogenic differentiation, lipid droplets were formed. Likewise, the amount of intracelluar triglycerides increased (**Supplementary Figures A–C**). Furthermore, the expression of adipogenic marker genes, e.g. PPARγ and Adiponectin, increased during adipogenesis on both, mRNA and protein level (**Supplementary Figures 2D, E**). Endogenous levels of miR-146a/b-5p in SGBS cells during adipogenesis remained largely stable (**Supplementary Figure 3**). Next, we treated SGBS adipocytes with trauma-relevant concentrations of IL-1β, IL-6, IL-8, C3a, and C5a either alone or in combination (referred to as ‘mix’). The mix of factors and the treatment with IL-1β alone induced an upregulation of IL-6 (gene: *IL6*), IL-8 (gene: *IL8*) and MCP1 (gene: *MCP1*) mRNA and protein expression (**Figures 2A, B**). The single treatment with either IL-6, IL-8, C3a, or C5a did not exert this effect (**Figures 2A, B**). This data suggests that, of the factors tested, IL-1β is the most potent inducer of inflammation in adipocytes. Of note, cell viability of SGBS adipocytes was neither affected by the interleukins or complement factors alone nor in the corresponding combination (**Figure 2C**). Along with the significant inflammatory response, miR-146a-5p was strongly induced by IL-1β or the mix of factors as early as 4 hours after exposure (**Figure 2D**). MiR-146b-5p was only significantly upregulated by stimulation with IL-1β after 24 hours (**Figure 2D**).

### Upregulation of miR-146a-5p by IL-1β is mediated *via* the NF-κB signaling pathway

**Figure 3:**
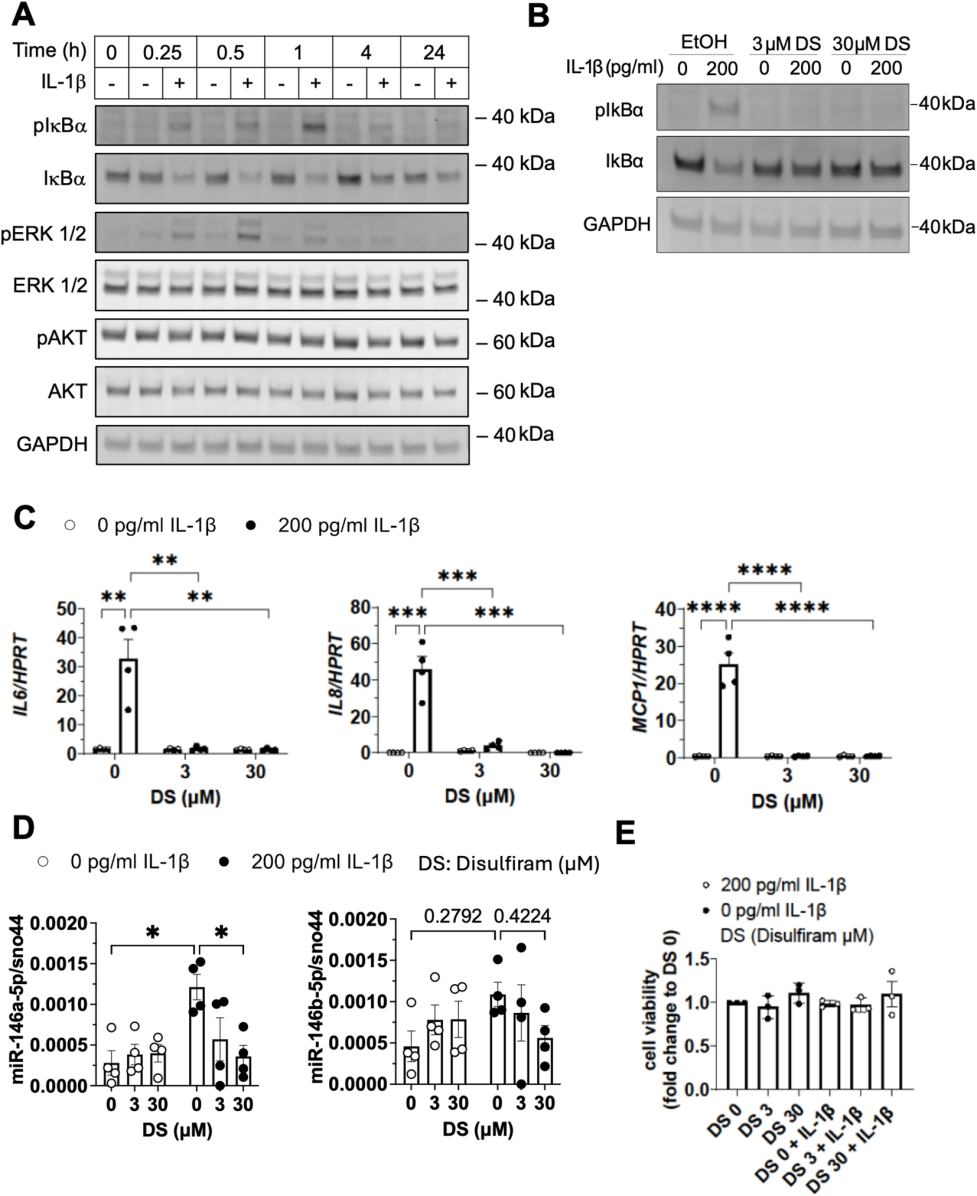
Upregulation of miR-146a-5p by IL-1β is mediated *via* the NF-κB signaling pathway. **(A)** SGBS adipocytes were treated with IL-1β (200 pg/ml) or the corresponding vehicle control. Protein was extracted 0, 0.25, 0.5, 1, 4, and 24 h post-stimulation. One representative Western Blot out of three is shown determining the protein expression of pIκBα, IκBα, pERK1/2, ERK1/2, pAKT, and AKT. GAPDH serves as loading control for the respective proteins. **(B–D)** SGBS adipocytes were treated with 3 µM, 30 µM DS or EtOH as control for 30 minutes before stimulation with IL-1β (200 pg/ml) or the corresponding vehicle control for 4 h. **(B)** Expression of pIκBα and IκBα was assessed by Western Blot with GAPDH as loading control. One representative Western Blot out of three independent experiments is shown. **(C)***IL6*, *IL8*, and *MCP1* mRNA expression levels were determined by RT-qPCR using the ΔCt-method with *HPRT* as reference gene. **(D)** miR-146a-5p and miR-146b-5p expression was assessed by RT-qPCR using the ΔCt-method with sno44 as reference gene. **(E)** SGBS adipocytes were treated with DS or EtOH as control in presence or absence of IL-1β (200 pg/ml) as indicated for 24 hours. Cell viability was measured by CellTiterGlo (CTG) assay. The results are displayed as mean ± SEM of three **(E)** or four **(C, D)** independent experiments performed in triplicates. Statistics: two-way ANOVA **(C, D)** or one-way [**(E)**, ns] with Tukey correction, *p < 0.05, **p < 0.01, p*** < 0.001, ****p < 0.0001.

Next, we assessed which downstream signaling pathways are activated by IL-1β in SGBS adipocytes. Here, we identified a robust induction of phosphorylation of IκBα (pIκBα) as early as 15 minutes after stimulation with IL-1β (**Figures 3A**, see **Supplementary Figure 4A** for quantification). Phosphorylation of IκBα is a prerequisite of its degradation and, indeed, protein levels of IκBα declined after IL-1β stimulation (**Figures 3A**, see **Supplementary Figure 4B** for quantification). IL-1β also induced phosphorylation of ERK (pERK) within one hour of stimulation while levels of total ERK protein were not affected (**Figures 3A**, see **Supplementary Figure 4C** for quantification). The AKT pathway was not activated by stimulation with IL-1β (**Figures 3A**, **Supplementary Figure 4D**). To further confirm that IL-1β stimulation leads to activation of the NF-κB pathway, we applied 3 and 30 µM of disulfiram (DS), a known NF-κB inhibitor (**Figures 3B, C**). We selected the concentrations in accordance with a previously published article (62). Indeed, co-incubation with DS prevented IL-1β-induced phosphorylation and degradation of IκBα (**Figure 3B**), and also strongly inhibited IL1β-mediated expression of inflammatory cytokines such *IL6*, *IL8*, and *MCP1* (**Figure 3C**). In line with our hypothesis that IL-1β leads to upregulation of miR-146a-5p via induction of NF-κB signaling, we found that the combination of IL-1β and DS prevented IL-1β-induced upregulation of miR-146a-5p (**Figure 3D**). Of note, cell viability of SGBS adipocytes was neither affected by DS alone nor by DS + IL-1β (**Figure 3E**).

For investigating the role of IL-1β on MAPK signaling, the MEK/ERK inhibitor trametinib was used. Trametinib prevented the IL-1β-induced phosphorylation of ERK1/2 (**Supplementary Figure 5A**) and significantly inhibited IL-1β-mediated induction of *IL6*, *IL8* and *MCP1* mRNA (**Supplementary Figure 5B**). Of note, cell viability of SGBS adipocytes remained unaffected by trametinib alone or in combination with IL-1β (**Supplementary Figure 5C**). Thus, the MEK/ERK signaling pathway contributes to IL-1β-mediated inflammation in human adipocytes.

### IL-1β-mediated inflammation is ameliorated by miR-146a-5p

**Figure 4:**
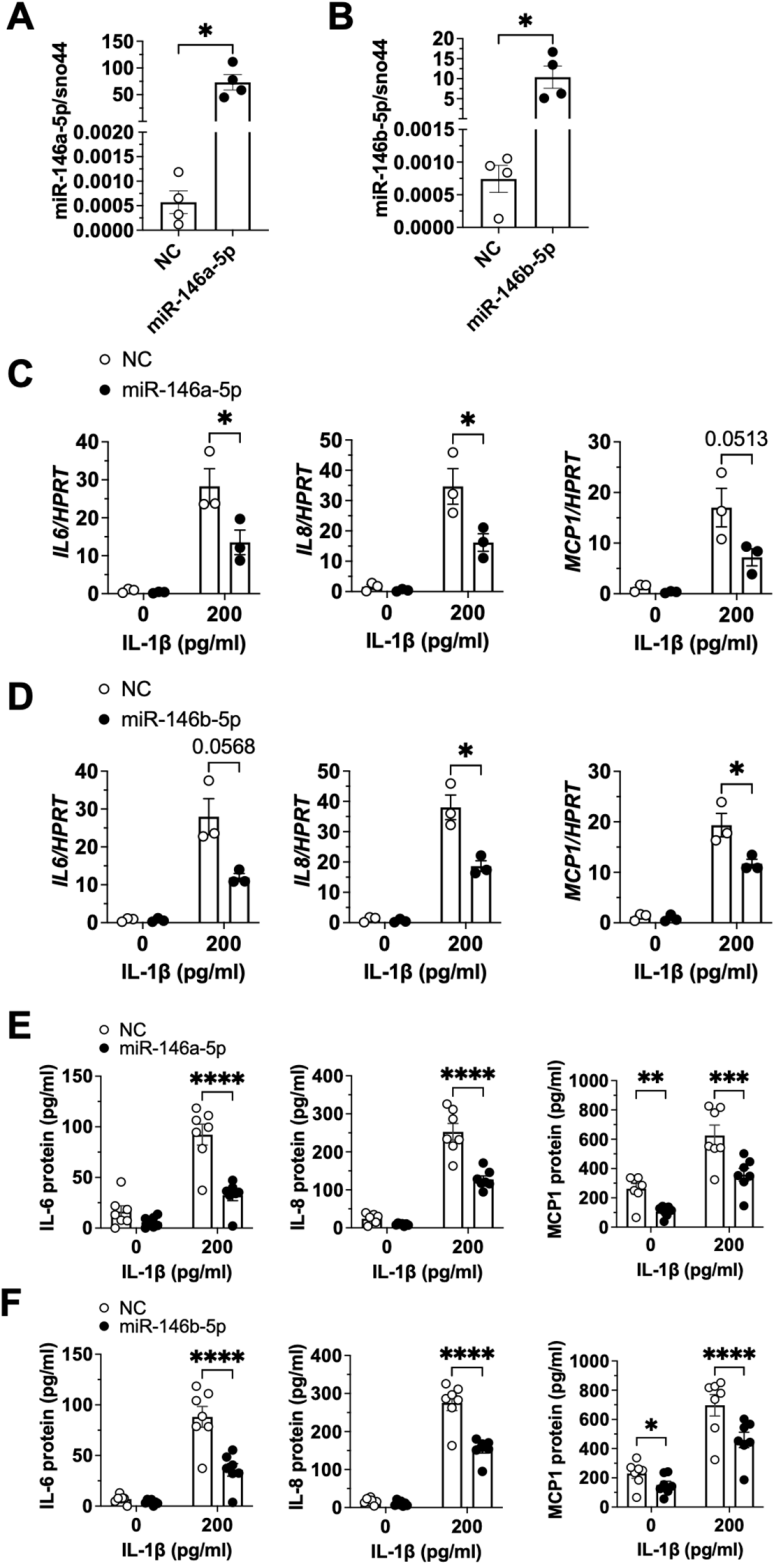
IL-1β-mediated inflammation is ameliorated by miR-146a-5p. SGBS adipocytes were transfected with 50 nM miR-146a-5p or miR-146b-5p mimic or a non-targeting control (NC). **(A, B)** Total RNA was isolated after 7 days. miR-146a-5p and miR-146b-5p expression levels were assessed by RT-qPCR using the ΔCt-method with sno44 as reference gene. The results are displayed as mean ± SEM of four independent experiments performed in triplicates. **(C–F)** SGBS adipocytes were stimulated with IL-1β (200 pg/ml) or the corresponding vehicle control 7 days post-transfection with miR-146a-5p mimic **(C, E)** or miR-146b-5p **(D, F)**. **(C, D)** Total RNA was isolated 4 h after stimulation. *IL6*, *IL8* and *MCP1* expression was assessed by RT-qPCR using ΔCt-method with *HPRT* as reference gene. The results are displayed as mean ± SEM of three independent experiments performed in triplicates. **(E, F)** IL-6, IL-8, and MCP1 were measured in media supernatants by ELISA after 4 hours of stimulation with IL-1β. The results are displayed as mean ± SEM of three independent experiments performed twice in triplicates and once individually. Statistics: paired two-tailed t-test **(A, B)**, two-way ANOVA with Šídák correction **(C–F)**, *p < 0.05, **p < 0.01, p*** < 0.001, ****p < 0.0001.

MiR-146a-5p and miR-146b-5p were upregulated in inguinal WAT depots after trauma (**Figures 1C, D**), and miR-146a-5p was upregulated in adipocytes following stimulation with IL-1β in a NF-κB-dependent manner *in vitro* (**Figures 2D**, **3D**). To further assess the impact of elevated levels of miR-146a-5p and miR-146b-5p on IL-1β-mediated inflammatory signaling in adipocytes, we transfected SGBS adipocytes with either miR-146a-5p or miR-146b-5p mimic (**Figures 4A, B**). Interestingly, both mimics dampened the IL-1β-induced inflammatory response on mRNA (**Figures 4C, D**) and protein level (**Figures 4E, F**). This suggests that both miRNAs can act as suppressors of IL-1β-induced proinflammatory signaling.

### miR-146a-5p suppresses IL-1β-induced proinflammatory signaling by downregulating IRAK1

**Figure 5:**
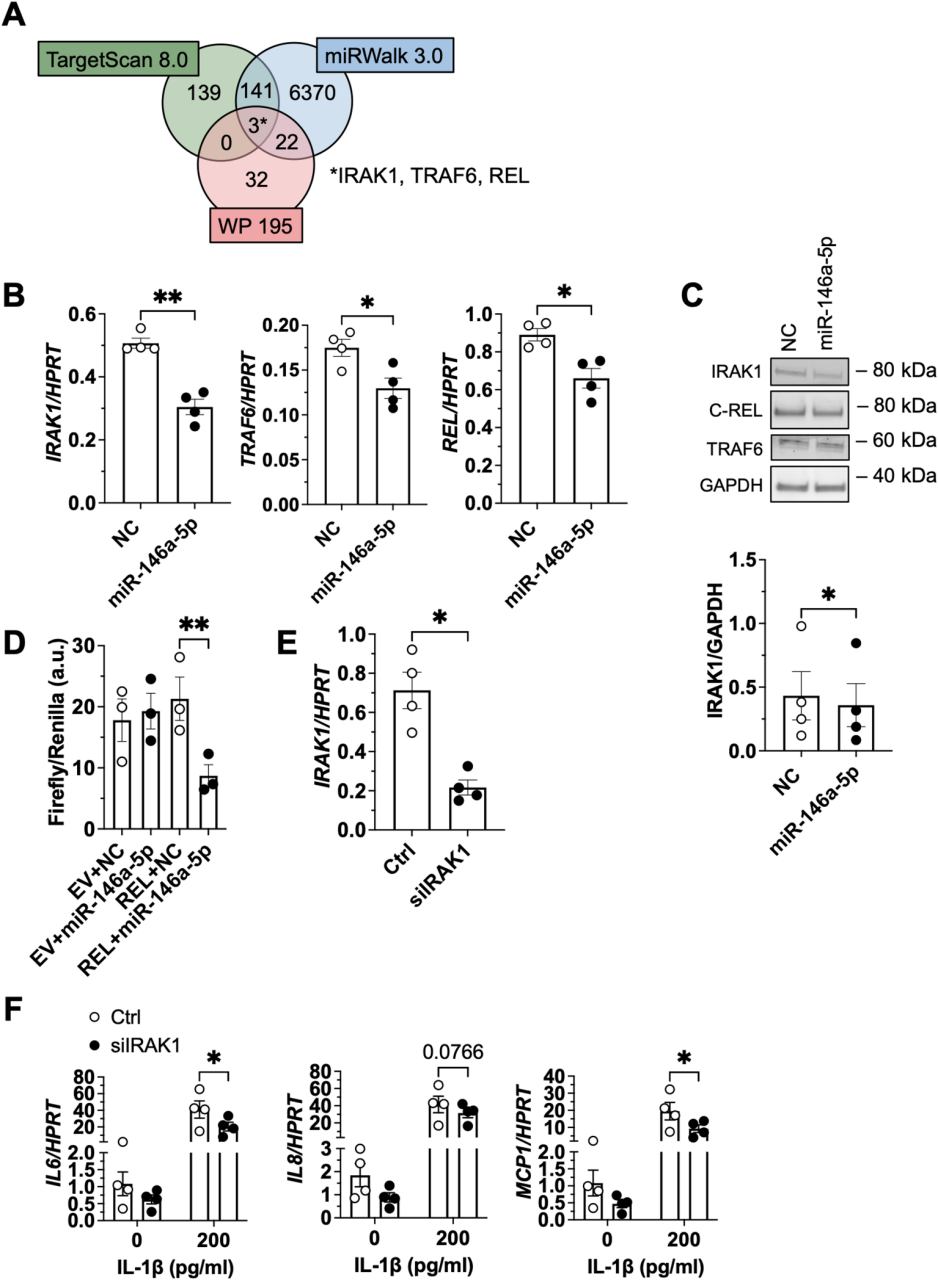
MiR-146a-5p suppresses IL-1β-induced proinflammatory signaling by targeting IRAK1 **(A)** Venn diagram representing the intersection of two *in silico* target gene predictions (TargetScan 8.0 and miRWalk 3.0) for miR-146a-5p and genes of the IL-1β signaling pathway (WikiPathway 195) resulting in an overlap of three genes, i.e. IRAK1, TRAF6, REL. **(B, C)** SGBS adipocytes were transfected with 50 nM miR-146a-5p mimic or a non-targeting control (NC). **(B)** Total RNA was isolated 7 days post-transfection. *IRAK1*, *TRAF6*, and *REL* mRNA levels were assessed by RT-qPCR using the ΔCt-method with *HPRT* as reference gene. The results are displayed as mean ± SEM of four independent experiments performed in triplicates. **(C)** Protein was extracted 7 days post-transfection. Protein expression of IRAK1, TRAF6 and c-REL was assessed by Western Blot with GAPDH as corresponding loading control. One representative Western Blot out of four independent experiments is shown. Densitometric analyses is displayed as mean ± SEM of four independent experiments performed in duplicates. **(D)** HEK293 cells were transfected as indicated (plasmid amount used: 25 ng, miRNA amount used: 100 nM). Next, a dual luciferase reporter gene assay was performed by determining the luciferase signal expressed as Firefly over Renilla, denoted in (a.u.). The results are displayed as mean ± SEM of three independent experiments performed in triplicates. **(E)** SGBS adipocytes were transfected with 20 nM IRAK1 siRNA or control (Ctrl). mRNA levels were assessed by RT-qPCR using the ΔCt-method with *HPRT* as reference gene 72 h post-transfection. The results are displayed as mean ± SEM of four independent experiments performed in triplicates. **(F)** siRNA-transfected SGBS adipocytes were stimulated with IL-1β (200 pg/ml) or the corresponding vehicle control 72 h post-transfection. Total RNA was isolated 4 h after stimulation. *IL6, IL8, and MCP1* mRNA expression was analyzed. Statistics: paired two-tailed t-test **(B, C, E)**, one-way ANOVA with Tukey correction, *p < 0.05, **p < 0.01 **(D)**, two-way ANOVA with Šídák correction, *p < 0.05 **(F)**.

To identify the underlying molecular mechanism of mitigation of IL-1β-induced signaling by miR-146a-5p and miR-146b-5p, we next predicted their target genes using TargetScan 8.0 and miRWalk 3.0 (35, 55). The predicted target genes were cross-referenced with those involved in IL-1β signaling (WikiPathway 195) resulting in three genes that matched both, the group of predicted targets and the genes involved in IL-1β signaling: *IRAK1*, *TRAF6* and *REL* (also known as c-REL) (miR-146a-5p: **Figure 5A**; miR-146b-5p: **Supplementary Figure 6A**) (37). To validate these predicted target genes, we transfected SGBS adipocytes with miR-146a-5p which resulted in significantly diminished mRNA expression of *IRAK1*, *TRAF6* and *REL* (**Figure 5B**). *IRAK1* mRNA was also significantly downregulated by miR-146b-5p whereas mRNA expression of *TRAF6* and *REL* was not significantly affected (**Supplementary Figure 6B**). Additional experiments confirmed significant downregulation of IRAK1, but not TRAF6 and REL (**Figure 5C**; **Supplementary Figure 6**) by miR-146a-5p on protein level, and direct targeting of REL by miR-146a-5p in a dual reporter gene assay (**Figure 5D**). As IRAK1 and TRAF6 are already well-known targets of miR-146a/b-5p, we did not further confirm it by a dual reporter gene assay (58). To determine whether IRAK1, TRAF6, and REL affected IL-1β-mediated proinflammatory signaling in adipocytes, we next performed knockdown experiments. First, siRNA knockdown efficiency was analyzed in adipocytes demonstrating a downregulation of IRAK1 (**Figure 5E**), TRAF6, and REL (**Supplementary Figure 6D**) on mRNA level. Next, adipocytes transfected with the respective siRNAs were stimulated with IL-1β, and the proinflammatory response of the cells was assessed by mRNA expression of *IL6*, *IL8*, and *MCP1* (**Figure 5F**; **Supplementary Figures 6E, F**). Interestingly, whereas the inflammatory response of adipocytes was not affected by the knockdown of TRAF6 or REL (**Supplementary Figures 6E, F**), knockdown of IRAK1 resulted in significantly reduced *IL6* and *MCP1* mRNA expression (**Figure 5F**).

Thus, miR-146a-5p, and potentially also miR-146b-5p, are upregulated by IL-1β in human adipocytes and downregulate IRAK1, a crucial kinase mediating proinflammatory signaling of IL-1β, thereby providing a negative feedback mechanism of adipocyte inflammation.

## Discussion

Adipose tissue is a major source of miRNAs which are known for their crucial role in the regulation of inflammatory signaling. Although adipose tissue is co-affected by almost any traumatic injury, it is understudied in the context of trauma (65). In this study, we investigated the role of miRNAs in inguinal WAT depots in a mouse model of PT+HS and identified miR-146a-5p and miR-146b-5p to be upregulated after trauma in inguinal WAT depots not directly affected by the traumatic force vector (**Figure 1**).

The mouse model used in this study represents a well-established murine model system of polytrauma combined with haemorrhagic shock involving thoracic trauma, closed head injury and femur fracture including soft tissue injury (63). Despite its wide acceptance in the field of polytrauma research, this model – like every other model – has limitations. For example, the mice in this study were anaesthesized with sevoflurane leading to unconsciousness, analgesia and muscle relaxation. Thus, the model does not fully recapitulate the acute nociceptive input, conscious perception and psychological stress of injury occurring in patients with polytrauma. As a consequence, the neuroendocrine stress pathways such as the hypothalamic-pituitary-adrenal axis and the sympathetic nervous system are not activated by pain. The absence of those neuroendocrine signals, in turn, can significantly influence immune cell activation and cytokine release (36). Moreover, anesthesia itself has immunomodulatory effects and may dampen both, catecholamine and glucocorticoid release modulating inflammatory or anti-inflammatory pathways, respectively (44).

miR-146a-5p and miR-146b-5p belong to the same miRNA family. The miR-146 family was first identified in mouse heart tissue in 2002, followed by the characterization of its genomic location and regulation in 2006 (28, 58). miR-146a and miR-146b are located on chromosome 5q33.3 and chromosome 10q24.32, respectively (42). MiR-146a-5p and miR-146b-5p are responsive to endotoxins and can be upregulated by proinflammatory factors (27, 58). In our model system of white adipocytes, IL-1β induced a proinflammatory response and, importantly, an upregulation of miR-146a-5p, and also miR-146b-5p although to a lesser extent (**Figure 2**). In addition to IL-1β, we also evaluated the effects of IL-6, IL-8, C3a, and C5a in adipocytes *in vitro.* These *c*ytokines and anaphylatoxins are often elevated in the plasma of polytrauma patients in the early phase after injury (4, 5, 8, 18, 22, 23, 50, 71). However, none of these factors induced an inflammatory response comparable to IL-1β in our model system *in vitro* (**Figure 2**) underlining the importance of IL-1β-induced inflammation in adipocytes. IL-1β activates NF-κB and MAPK signaling pathways leading to the production and secretion of proinflammatory mediators (1, 2, 6, 40, 54). In this study, we demonstrated that IL-1β triggers the upregulation of miR-146a-5p in human adipocytes via the NF-κB signaling pathway, which was suppressed when the NF-κB signaling pathway was blocked by Disulfiram (**Figure 3D**). This is in line with previous studies reporting NF-κB-regulated transcription of miR-146a-5p (31, 41, 58).

Both, miR-146a-5p and miR-146b-5p are highly conserved across species and differ in only two nucleotides at the 3’-end of the mature strand, but not within the seed region (42). Thus, it is not surprising that we observed similar effects of these two miRNAs on IL-1β signaling in our study in human white adipocytes (**Figure 4**). However, miR-146a-5p and miR-146b-5p were also shown to possess unique regulatory functions (42). Previous publications showed that IL-1β induced a stronger upregulation of miR-146a-5p than miR-146b-5p (7, 43), in line with our results (**Figure 2D**). Furthermore, it was shown that miR-146a-5p and miR-146b-5p are differentially regulated in peripheral blood mononuclear cells in patients with chronic kidney disease (68) illustrating that these two miRNAs are not necessarily regulated in the same manner. Importantly both, miR-146a-5p and miR-146b-5p, inhibited the IL-1β-induced proinflammatory response of SGBS adipocytes *in vitro* (**Figures 4C–F**), and can, thus, be regarded as anti-inflammatory factors ameliorating the trauma response of adipocytes. Of note, miR-146a-5p and miR-146b-5p modulate the response to IL-1β as negative feedback regulators rather than influencing IL-1β production and release. Also in other model systems, miR-146a-5p and miR-146b-5p were shown to inhibit IL-1β-mediated proinflammatory signaling (7, 25, 39, 41, 43, 47).

In adipocytes, IL-1β can trigger a proinflammatory response leading to impaired insulin sensitivity (3, 17, 57, 64). Importantly, we and others found miR-146a-5p to be elevated under inflammatory conditions in the obese tissue of mice and humans (47, 51). MiR-146a plays an important role in insulin sensitivity as miR146^-/-^ mice showed increased insulin resistance on high fat diet as compared to control mice (46). Hyperglycemia and insulin resistance often occur in patients with polytrauma (30). IL-1β was upregulated in preclinical studies of polytrauma (22, 59). This suggests that trauma patients with increased levels of IL-1β may benefit from a treatment with anakinra, a human IL-1 receptor antagonist, to maintain metabolic health (22, 59). Indeed, anakinra reduced inflammation and tissue injury in an experimental rat model of traumatic brain injury (20). This is in line with data from preclinical models of traumatic brain injury, in which blockage of IL-1β showed signs of neuroprotection (59).

Using an *in silico* target gene prediction tool, we identified *IRAK1*, *TRAF6*, and *REL* as potential target genes of miR-146a-5p and miR-146b-5p. All three genes are involved in the IL-1β signaling pathway. Using siRNA-mediated knockdown, we could show that IRAK1 is a crucial mediator of the IL-1β-induced inflammatory response (**Figure 5F**). IRAK1 is a well-established target of miR-146a/b-5p as demonstrated by luciferase reporter assays in a number of studies across different cell lines including HEK cells, HUVEC cells and neuronal PC12 cells in the past (11, 58, 67). We confirmed *IRAK1* to be downregulated by miR-146a-5p in human adipocytes (**Figures 5B, C**). As REL was not previously studied in the context of miR-146a-5p function, we decided to perform a dual-luciferase reporter assay in HEK cells and identified *REL* as a direct target (**Figure 5D**). However, transfection with miR-146a-5p resulted in downregulation of *REL* on mRNA but not on protein level (**Figures 5B, C**). Comparable results were found for *TRAF6*, another well-known target gene of miR-146a-5p (58) (**Figures 5B, C**). Loss-of-function experiments revealed that downregulation of *IRAK1* significantly reduced the inflammatory effect of IL-1β (**Figure 5F**), while down-regulation of *TRAF6* or *REL* did not show this effect (**Supplementary Figures 6E, F**). Therefore we conclude that downregulation of IRAK1 is the relevant step of miR-146a-5p dampening the IL-1β-mediated inflammatory signaling. Of note, the effects of IRAK1 siRNA were significant but did not reach the effect size of miR-146a-5p mimic transfection. We therefore propose that miR-146a-5p acts via additional target genes and pathways involved in inflammation. This is in line with the fact that one miRNA can regulate several hundreds of target genes at the same time (10).

Adipose tissue contributes to exacerbation of the systemic inflammatory response to trauma (65). Prevention of systemic inflammation and its detrimental sequelae in patients with polytrauma requires early identification of patients at increased risk. This could be accomplished through the identification of biomarkers that reflect trauma severity, thereby aiding in the monitoring and management of polytraumatized patients. We show that the miRNAs miR-146a-5p and miR-146b-5p are differentially expressed in WAT after polytrauma. In general, miRNAs are good candidates for biomarkers as (i) they are stable in various body fluids, (ii) the expression of some miRNAs is tissue-specific, and (iii) the expression level of miRNAs can be easily measured using various methods (13, 45). In addition, miRNAs can be packaged into exosomes and are, thus, found in extracellular body fluids (16, 60, 70). In line, miR-146a-5p was significantly upregulated in plasma of patients with polytrauma (**Figure 1E**, (GSE223151) (56)). Whether miR-146a-5p and miR-146b-5p are secreted from WAT by being packed into exosomes is currently unclear. As both miRNAs suppress the proinflammatory signaling in WAT mediated by IL-1β their levels could correlate with a beneficial outcome in patients with polytrauma. Importantly, circulating miRNA levels do not necessarily represent the functional intracellular concentrations within relevant cells or tissues. An increase in circulating miRNA levels may result from passive release from damaged or dying cells. As such, elevated plasma levels do not necessarily indicate sufficient or sustained activity at the cellular or tissue level (69). It is therefore plausible that miR-146a/b-5p activity is transient, spatially limited, or quantitatively insufficient. Additional augmentation using miRNA mimics could provide further therapeutic benefit by enhancing intracellular miRNA availability in critical cell populations involved in inflammatory signaling. Given that miR-146a-5p and miR-146b-5p dampen IL-1β-mediated proinflammatory pathways in WAT, increasing their activity therapeutically could contribute to a beneficial outcome in polytraumatized patients. A limitation of our study is that we only provide data from *in vitro* cytokine-stimulation experiments and correlation analyses. Further experiments will be required to provide *in vivo* evidence that therapeutic delivery of miR-146 is effective in the context of polytrauma.

Taken together, our study identified miR-146a-5p and miR-146b-5p as trauma-relevant miRNAs that are regulators of the inflammatory response of WAT to polytrauma. We discovered IL-1β as crucial mediator of an inflammatory response in human white adipocytes, and found that miR-146a-5p and miR-146b-5p dampen the IL-1β-mediated inflammatory signaling by downregulating IRAK1. Since miR-146a-5p was upregulated in plasma of polytrauma patients, it is tempting to speculate that it could potentially serve as biomarker to monitor the body’s response to polytrauma in the future.

## Data Availability

The datasets presented in this study can be found in online repositories. The names of the repository/repositories and accession number(s) can be found below: GSE302289 (GEO).

## References

[1] Agostini L Martinon F Burns K McDermott MF Hawkins PN Tschopp J . NALP3 Forms an IL-1β-Processing Inflammasome with Increased Activity in Muckle-Wells Autoinflammatory Disorder. Immunity. (2004) 20:319–25. doi: 10.1016/S1074-7613(04)00046-9, PMID: 15030775

[2] Berg AH Lin Y Lisanti MP Scherer PE . Adipocyte differentiation induces dynamic changes in NF-κB expression and activity. Am J Physiology-Endocrinol Metab. (2004) 287:E1178–88. doi: 10.1152/ajpendo.00002.2004, PMID: 15251865

[3] Bing C . Is interleukin-1β a culprit in macrophage-adipocyte crosstalk in obesity? Adipocyte. (2015) 4:149–52. doi: 10.4161/21623945.2014.979661, PMID: 26167419 PMC4496963

[4] Bogner V Keil L Kanz K-G Kirchhoff C Leidel B Mutschler W . Very early posttraumatic serum alterations are significantly associated to initial massive RBC substitution, injury severity, multiple organ failure and adverse clinical outcome in multiple injured patients. Eur J Med Res. (2009) 14:284. doi: 10.1186/2047-783X-14-7-284, PMID: 19661010 PMC3458638

[5] Burk A-M Martin M Flierl MA Rittirsch D Helm M Lampl L . Early Complementopathy After Multiple Injuries in Humans. Shock. (2012) 37:348–54. doi: 10.1097/SHK.0b013e3182471795, PMID: 22258234 PMC3306539

[6] Chen GY Nuñez G . Sterile inflammation: sensing and reacting to damage. Nat Rev Immunol. (2010) 10:826–37. doi: 10.1038/nri2873, PMID: 21088683 PMC3114424

[7] Cheng HS Sivachandran N Lau A Boudreau E Zhao JL Baltimore D . Micro RNA -146 represses endothelial activation by inhibiting pro-inflammatory pathways. EMBO Mol Med. (2013) 5:1017–34. doi: 10.1002/emmm.201202318, PMID: 23733368 PMC3721471

[8] Chiaretti A Genovese O Aloe L Antonelli A Piastra M Polidori G . Interleukin 1β and interleukin 6 relationship with paediatric head trauma severity and outcome. Childs Nerv Syst. (2005) 21:185–93. doi: 10.1007/s00381-004-1032-1, PMID: 15455248

[9] Denk S Weckbach S Eisele P Braun CK Wiegner R Ohmann JJ . Role of Hemorrhagic Shock in Experimental Polytrauma. Shock. (2018) 49:154–63. doi: 10.1097/SHK.0000000000000925, PMID: 28614141

[10] Diener C Keller A Meese E . The miRNA–target interactions: An underestimated intricacy. Nucleic Acids Res. (2024) 52:1544–57. doi: 10.1093/nar/gkad1142, PMID: 38033323 PMC10899768

[11] Echavarria R Mayaki D Neel J-C Harel S Sanchez V Hussain SNA . Angiopoietin-1 inhibits toll-like receptor 4 signalling in cultured endothelial cells: role of miR-146b-5p. Cardiovasc Res. (2015) 106:465–77. doi: 10.1093/cvr/cvv120, PMID: 25824148

[12] Elabd C Chiellini C Carmona M Galitzky J Cochet O Petersen R . Human Multipotent Adipose-Derived Stem Cells Differentiate into Functional Brown Adipocytes. Stem Cells. (2009) 27:2753–60. doi: 10.1002/stem.200, PMID: 19697348

[13] Etheridge A Lee I Hood L Galas D Wang K . Extracellular microRNA: A new source of biomarkers. Mutat Research/Fundamental Mol Mech Mutagen. (2011) 717:85–90. doi: 10.1016/j.mrfmmm.2011.03.004, PMID: 21402084 PMC3199035

[14] Filipowicz W Bhattacharyya SN Sonenberg N . Mechanisms of post-transcriptional regulation by microRNAs: are the answers in sight? Nat Rev Genet. (2008) 9:102–14. doi: 10.1038/nrg2290, PMID: 18197166

[15] Fischer-Posovszky P Newell FS Wabitsch M Tornqvist HE . Human SGBS Cells &ndash; a Unique Tool for Studies of Human Fat Cell Biology. Obes Facts. (2008) 1:184–9. doi: 10.1159/000145784, PMID: 20054179 PMC6452113

[16] Foessl I Haudum CW Vidakovic I Prassl R Franz J Mautner SI . miRNAs as Regulators of the Early Local Response to Burn Injuries. IJMS. (2021) 22:9209. doi: 10.3390/ijms22179209, PMID: 34502118 PMC8430593

[17] Gao D Madi M Ding C Fok M Steele T Ford C . Interleukin-1β mediates macrophage-induced impairment of insulin signaling in human primary adipocytes. Am J Physiology-Endocrinol Metab. (2014) 307:E289–304. doi: 10.1152/ajpendo.00430.2013, PMID: 24918199 PMC4121578

[18] Giannoudis PV Smith MR Evans RT Bellamy MC Guillou PJ . Serum CRP and IL-6 levels after trauma: Not predictive of septic complications in 31 patients. Acta Orthopaed Scandinav. (1998) 69:184–8. doi: 10.3109/17453679809117625, PMID: 9602781

[19] Harrison PW Amode MR Austine-Orimoloye O Azov AG Barba M Barnes I . Ensembl 2024. Nucleic Acids Res. (2024) 52:D891–9. doi: 10.1093/nar/gkad1049, PMID: 37953337 PMC10767893

[20] Hasturk AE Yilmaz ER Turkoglu E Kertmen H Horasanli B Hayirli N . Therapeutic evaluation of interleukin 1-beta antagonist Anakinra against traumatic brain injury in rats. Ulus Travma Acil Cerrahi Derg. (2015) 21:1–8. doi: 10.5505/tjtes.2015.57894, PMID: 25779705

[21] Haupt J Krysiak N Unger M Bogner-Flatz V Biberthaler P Hanschen M . The potential of adipokines in identifying multiple trauma patients at risk of developing multiple organ dysfunction syndrome. Eur J Med Res. (2021) 26:38. doi: 10.1186/s40001-021-00511-z, PMID: 33931112 PMC8086117

[22] Hengartner N-E Fiedler J Schrezenmeier H Huber-Lang M Brenner RE . Crucial Role of IL1beta and C3a in the *In Vitro*-Response of Multipotent Mesenchymal Stromal Cells to Inflammatory Mediators of Polytrauma. PloS One. (2015) 10:e0116772. doi: 10.1371/journal.pone.0116772, PMID: 25562599 PMC4285554

[23] Hensler T Heinemann B Sauerland S Lefering R Bouillon B Andermahr J . Immunologic Alterations Associated with High Blood Transfusion Volume After Multiple Injury: Effects on Plasmatic Cytokine and Cytokine Receptor Concentrations. Shock (2003) 20:497–502. doi: 10.1097/01.shk.0000095058.62263.1f, PMID: 14625472

[24] Huber-Lang M Lambris JD Ward PA . Innate immune responses to trauma. Nat Immunol. (2018) 19:327–41. doi: 10.1038/s41590-018-0064-8, PMID: 29507356 PMC6027646

[25] Iyer A Zurolo E Prabowo A Fluiter K Spliet WGM van Rijen PC . MicroRNA-146a: A Key Regulator of Astrocyte-Mediated Inflammatory Response. PloS One. (2012) 7:e44789. doi: 10.1371/journal.pone.0044789, PMID: 23028621 PMC3441440

[26] Jung U Choi M-S . Obesity and Its Metabolic Complications: The Role of Adipokines and the Relationship between Obesity, Inflammation, Insulin Resistance, Dyslipidemia and Nonalcoholic Fatty Liver Disease. IJMS. (2014) 15:6184–223. doi: 10.3390/ijms15046184, PMID: 24733068 PMC4013623

[27] Kutty RK Nagineni CN Samuel W Vijayasarathy C Jaworski C Duncan T . Differential regulation of microRNA-146a and microRNA-146b-5p in human retinal pigment epithelial cells by interleukin-1β, tumor necrosis factor-α, and interferon-γ. Mol Vis. (2013) 19:737–50. PMC3626297 23592910

[28] Lagos-Quintana M Rauhut R Yalcin A Meyer J Lendeckel W Tuschl T . Identification of Tissue-Specific MicroRNAs from Mouse. Curr Biol. (2002) 12:735–9. doi: 10.1016/S0960-9822(02)00809-6, PMID: 12007417

[29] Lei P Li Y Chen X Yang S Zhang J . Microarray based analysis of microRNA expression in rat cerebral cortex after traumatic brain injury. Brain Res. (2009) 1284:191–201. doi: 10.1016/j.brainres.2009.05.074, PMID: 19501075

[30] Li L Messina JL . Acute insulin resistance following injury. Trends Endocrinol Metab. (2009) 20:429–35. doi: 10.1016/j.tem.2009.06.004, PMID: 19800814 PMC2939005

[31] Li YY Cui JG Hill JM Bhattacharjee S Zhao Y Lukiw WJ . Increased expression of miRNA-146a in Alzheimer’s disease transgenic mouse models. Neurosci Lett. (2011) 487:94–8. doi: 10.1016/j.neulet.2010.09.079, PMID: 20934487 PMC5382794

[32] Livak KJ Schmittgen TD . Analysis of Relative Gene Expression Data Using Real-Time Quantitative PCR and the 2–ΔΔCT Method. Methods. (2001) 25:402–8. doi: 10.1006/meth.2001.1262, PMID: 11846609

[33] LLC . GB SnapGene | Software for everyday molecular biology. Available online at: https://www.snapgene.com/ (Accessed November 24, 2024).

[34] Luís A Hackl M Jafarmadar M Keibl C Jilge JM Grillari J . Circulating miRNAs Associated With ER Stress and Organ Damage in a Preclinical Model of Trauma Hemorrhagic Shock. Front Med. (2020) 7:568096. doi: 10.3389/fmed.2020.568096, PMID: 33072784 PMC7542230

[35] Witkos T M Koscianska E J Krzyzosiak W . Practical Aspects of microRNA Target Prediction. CMM. (2011) 11:93–109. doi: 10.2174/156652411794859250, PMID: 21342132 PMC3182075

[36] Maddali S Stapleton PP Freeman TA Smyth GP Duff M Yan Z . Neuroendocrine responses mediate macrophage function after trauma. Surgery. (2004) 136:1038–46. doi: 10.1016/j.surg.2004.03.001, PMID: 15523398

[37] Martens M Ammar A Riutta A Waagmeester A Slenter DN Hanspers K . WikiPathways: connecting communities. Nucleic Acids Res. (2021) 49:D613–21. doi: 10.1093/nar/gkaa1024, PMID: 33211851 PMC7779061

[38] McGeary SE Lin KS Shi CY Pham TM Bisaria N Kelley GM . The biochemical basis of microRNA targeting efficacy. Science. (2019) 366:eaav1741. doi: 10.1126/science.aav1741, PMID: 31806698 PMC7051167

[39] Meisgen F Xu Landén N Wang A Réthi B Bouez C Zuccolo M . MiR-146a Negatively Regulates TLR2-Induced Inflammatory Responses in Keratinocytes. J Invest Dermatol. (2014) 134:1931–40. doi: 10.1038/jid.2014.89, PMID: 24670381

[40] O’Neill LAJ . The interleukin-1 receptor/Toll-like receptor superfamily: 10 years of progress. Immunol Rev. (2008) 226:10–8. doi: 10.1111/j.1600-065X.2008.00701.x, PMID: 19161412

[41] Pan J Du M Cao Z Zhang C Hao Y Zhu J . miR-146a-5p attenuates IL-1β-induced IL-6 and IL-1β expression in a cementoblast-derived cell line. Oral Dis. (2020) 26:1308–17. doi: 10.1111/odi.13333, PMID: 32176411

[42] Paterson MR Kriegel AJ . MiR-146a/b: a family with shared seeds and different roots. Physiol Genomics. (2017) 49:243–52. doi: 10.1152/physiolgenomics.00133.2016, PMID: 28213571 PMC5407182

[43] Perry MM Moschos SA Williams AE Shepherd NJ Larner-Svensson HM Lindsay MA . Rapid Changes in MicroRNA-146a Expression Negatively Regulate the IL-1β-Induced Inflammatory Response in Human Lung Alveolar Epithelial Cells. J Immunol. (2008) 180:5689–98. doi: 10.4049/jimmunol.180.8.5689, PMID: 18390754 PMC2639646

[44] Reysner T Wieczorowska-Tobis K Kowalski G Grochowicka M Pyszczorska M Mularski A . The Influence of Regional Anesthesia on the Systemic Stress Response. Reports. (2024) 7:89. doi: 10.3390/reports7040089, PMID: 40757696 PMC12199975

[45] Rogobete AF Sandesc D Bedreag OH Papurica M Popovici SE Bratu T . MicroRNA Expression is Associated with Sepsis Disorders in Critically Ill Polytrauma Patients. Cells. (2018) 7:271. doi: 10.3390/cells7120271, PMID: 30551680 PMC6316368

[46] Roos J Dahlhaus M Funcke JB Kustermann M Strauss G Halbgebauer D . miR-146a regulates insulin sensitivity via NPR3. Cell MolLife Sci. (2021) 78:2987–3003. doi: 10.1007/s00018-020-03699-1, PMID: 33206203 PMC8004521

[47] Roos J Enlund E Funcke JB Tews D Holzmann K Debatin KM . miR-146a-mediated suppression of the inflammatory response in human adipocytes. SciRep. (2016) 6:38339. doi: 10.1038/srep38339, PMID: 27922090 PMC5138634

[48] Roos J Zinngrebe J Huber-Lang M Lupu L Schmidt MA Strobel H . Trauma-associated extracellular histones mediate inflammation via a MYD88-IRAK1-ERK signaling axis and induce lytic cell death in human adipocytes. Cell Death Dis. (2024) 15:285. doi: 10.1038/s41419-024-06676-9, PMID: 38653969 PMC11039744

[49] Rosen ED Spiegelman BM . What We Talk About When We Talk About Fat. Cell. (2014) 156:20–44. doi: 10.1016/j.cell.2013.12.012, PMID: 24439368 PMC3934003

[50] Roumen RMH Hendriks T van der Ven-Jongekrijg J Nieuwenhuijzen GAP Sauerwein RW van der Meer JWM . Cytokine Patterns in Patients After Major Vascular Surgery, Hemorrhagic Shock, and Severe Blunt Trauma Relation with Subsequent Adult Respiratory Distress Syndrome and Multiple Organ Failure. Ann Surg. (1993) 218:769–76. doi: 10.1097/00000658-199312000-00011, PMID: 8257227 PMC1243073

[51] Russo A Bartolini D Mensà E Torquato P Albertini MC Olivieri F . Physical Activity Modulates the Overexpression of the Inflammatory miR-146a-5p in Obese Patients: miR-146a-5p AND PHYSICAL ACTIVITY IN OBESITY. IUBMB Life. (2018) 70:1012–22. doi: 10.1002/iub.1926, PMID: 30212608

[52] Saliminejad K Khorram Khorshid HR Soleymani Fard S Ghaffari SH . An overview of microRNAs: Biology, functions, therapeutics, and analysis methods. J Cell Physiol. (2019) 234:5451–65. doi: 10.1002/jcp.27486, PMID: 30471116

[53] Shukla GC Singh J Barik S . MicroRNAs: Processing, Maturation, Target Recognition and Regulatory Functions. Mol Cell Pharmacol. (2011) 3:83. 22468167 PMC3315687

[54] Sims JE Smith DE . The IL-1 family: regulators of immunity. Nat Rev Immunol. (2010) 10:89–102. doi: 10.1038/nri2691, PMID: 20081871

[55] Sticht C de la Torre C Parveen A Gretz N . miRWalk: An online resource for prediction of microRNA binding sites. PloS One. (2018) 13:e0206239. doi: 10.1371/journal.pone.0206239, PMID: 30335862 PMC6193719

[56] Suen AO Chen F Wang S Li Z Zhu J Yang Y . Extracellular RNA Sensing Mediates Inflammation and Organ Injury in a Murine Model of Polytrauma. J Immunol. (2023) 210:1990–2000. doi: 10.4049/jimmunol.2300103, PMID: 37133342 PMC10235856

[57] Tack CJ Stienstra R Joosten LAB Netea MG . Inflammation links excess fat to insulin resistance: the role of the interleukin-1 family. Immunol Rev. (2012) 249:239–52. doi: 10.1111/j.1600-065X.2012.01145.x, PMID: 22889226

[58] Taganov KD Boldin MP Chang K-J Baltimore D . NF-κB-dependent induction of microRNA miR-146, an inhibitor targeted to signaling proteins of innate immune responses. Proc Natl Acad Sci USA. (2006) 103:12481–6. doi: 10.1073/pnas.0605298103, PMID: 16885212 PMC1567904

[59] Thome JG Reeder EL Collins SM Gopalan P Robson MJ . Contributions of Interleukin-1 Receptor Signaling in Traumatic Brain Injury. Front Behav Neurosci. (2020) 13:287. doi: 10.3389/fnbeh.2019.00287, PMID: 32038189 PMC6985078

[60] Thomou T Mori MA Dreyfuss JM Konishi M Sakaguchi M Wolfrum C . Adipose-derived circulating miRNAs regulate gene expression in other tissues. Nature. (2017) 542:450–5. doi: 10.1038/nature21365, PMID: 28199304 PMC5330251

[61] Wabitsch M Brenner R Melzner I Braun M Möller P Heinze E . Characterization of a human preadipocyte cell strain with high capacity for adipose differentiation. Int J Obes. (2001) 25:8–15. doi: 10.1038/sj.ijo.0801520, PMID: 11244452

[62] Wang W McLeod HL Cassidy J . Disulfiram-mediated inhibition of NF-?B activity enhances cytotoxicity of 5-fluorouracil in human colorectal cancer cell lines. Int J Cancer. (2003) 104:504–11. doi: 10.1002/ijc.10972, PMID: 12584750

[63] Weckbach S Hohmann C Braumueller S Denk S Klohs B Stahel PF . Inflammatory and apoptotic alterations in serum and injured tissue after experimental polytrauma in mice: distinct early response compared with single trauma or “double-hit” injury. J Trauma Acute Care Surg. (2013) 74:489–98. doi: 10.1097/TA.0b013e31827d5f1b, PMID: 23354243

[64] Wen H Gris D Lei Y Jha S Zhang L Huang MT-H . Fatty acid–induced NLRP3-ASC inflammasome activation interferes with insulin signaling. Nat Immunol. (2011) 12:408–15. doi: 10.1038/ni.2022, PMID: 21478880 PMC4090391

[65] Wrba L Halbgebauer R Roos J Huber-Lang M Fischer-Posovszky P . Adipose tissue: a neglected organ in the response to severe trauma? Cell Mol Life Sci. (2022) 79:207. doi: 10.1007/s00018-022-04234-0, PMID: 35338424 PMC8956559

[66] Wrba L Ohmann JJ Eisele P Chakraborty S Braumüller S Braun CK . Remote Intestinal Injury Early After Experimental Polytrauma and Hemorrhagic Shock. Shock. (2019) 52:e45–51. doi: 10.1097/SHK.0000000000001271, PMID: 30289852

[67] Yang G Zhao Y . Overexpression of miR-146b-5p Ameliorates Neonatal Hypoxic Ischemic Encephalopathy by Inhibiting IRAK1/TRAF6/TAK1/NF-αB Signaling. Yonsei Med J. (2020) 61:660. doi: 10.3349/ymj.2020.61.8.660, PMID: 32734729 PMC7393297

[68] Zawada AM Rogacev KS Müller S Rotter B Winter P Fliser D . Massive analysis of cDNA Ends (MACE) and miRNA expression profiling identifies proatherogenic pathways in chronic kidney disease. Epigenetics. (2014) 9:161–72. doi: 10.4161/epi.26931, PMID: 24184689 PMC3928179

[69] Zhao C Sun X Li L . Biogenesis and function of extracellular miRNAs. ExRNA. (2019) 1:38. doi: 10.1186/s41544-019-0039-4, PMID: 41762274

[70] Zhu H Fan G-C . Extracellular/circulating microRNAs and their potential role in cardiovascular disease. Am J Cardiovasc Dis. (2011) 1:138–49. PMC3207246 22059153

[71] Zilow G Sturm JA Rother U Kirschfink M . Complement activation and the prognostic value of C3a in patients at risk of adult respiratory distress syndrome. Clin Exp Immunol. (2008) 79:151–7. doi: 10.1111/j.1365-2249.1990.tb05171.x, PMID: 2311295 PMC1534766

[72] Deutsche Gesellschaft für Unfallchirurgie e . V.: S3-Leitlinie Polytrauma/Schwerverletzten-Behandlung (AWMF Registernummer 187-023), Version 4.0 (31.12.2022). Available online at: https://www.awmf.org/leitlinien/detail/ll/187-023.html (Accessed February 28, 2023).

[73] Transcript: ENST00000295025.12 (REL-201) - cDNA sequence - Homo_sapiens - Ensembl genome browser. Available online at: https://www.ensembl.org/Homo_sapiens/Transcript/Sequence_cDNA?db=core;g=ENSG00000162924;r=2:60881491-60931612;t=ENST00000295025 (Accessed November 24, 2024).

