# Supplementary material for: Upregulation of miR-146a-5p and miR-146b-5p limits IL-1β-mediated signaling in adipose tissue during polytrauma by downregulating IRAK1
Source: Front Immunol. 2026 Mar 4;17:1658504. doi: 10.3389/fimmu.2026.1658504 (PMC12995679; doi:10.3389/fimmu.2026.1658504)
Supplement: Supplementary file 1 [file DataSheet1.pdf]

Supplementary Information

Upregulation of miR-146a-5p and miR-146b-5p limits IL-1β-mediated signaling in adipose tissue during polytrauma by downregulating IRAK1

Antonia Mortsch [1], Julian Roos [1], Rebecca Halbgebauer [2], Ludmila Lupu [2], Annette Palmer [2], Anja Werberger [1], Ulrich Stifel [1], Martin Wabitsch [3,4], Markus Huber-Lang [2], Julia Zinngrebe [1], Pamela Fischer-Posovszky [1,4,#]

[1] Department of Pediatrics and Adolescent Medicine, Ulm University Medical Centre, Ulm, Germany

[2] Institute of Clinical and Experimental Trauma Immunology, Ulm University Medical Centre, Ulm, Germany

[3] Department of Pediatrics and Adolescent Medicine, Division of Pediatric Endocrinology and Diabetes, Ulm University Medical Centre, Ulm, Germany

[4] German Center for Child and Adolescent Health (DZKJ), partner site Ulm, Ulm, Germany

[#]Corresponding author: Pamela Fischer-Posovszky (pamela.fischer@uniklinik-ulm.de)

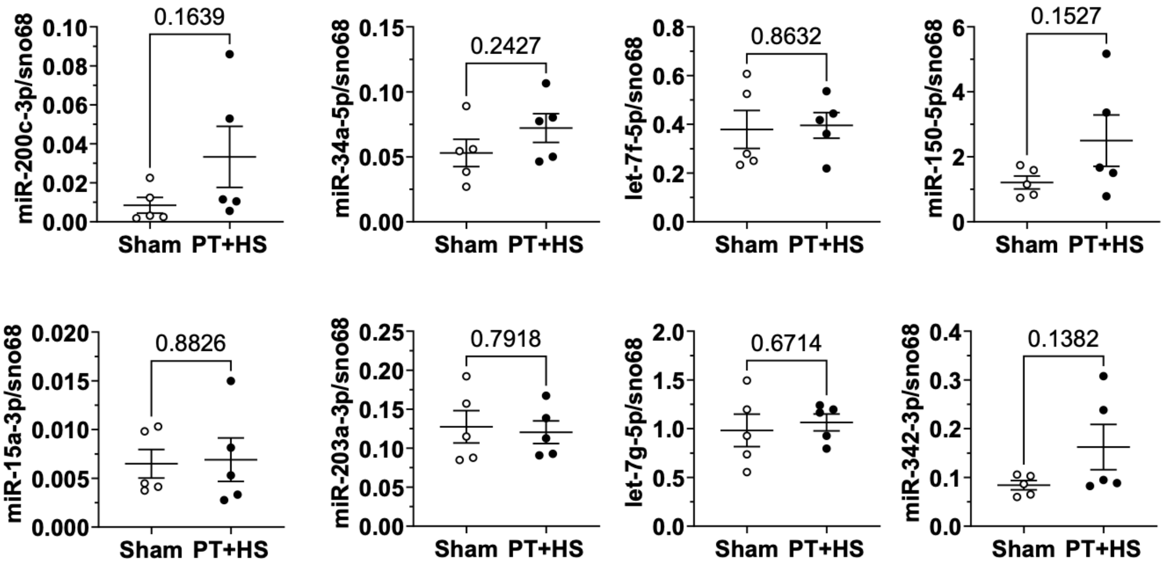

Supplementary Figure S1. Validation of miRNA microarray data by qRT-PCR. Differentially expressed miRNAs conserved between human and mouse, with a p-value ≤ 0.05 and a fold change (FC) of ≥ 2.0 for PT+HS versus sham in iWAT identified in the miRNA microarray were validated by qRT-PCR using the ΔCt-method with sno68 as reference gene. The results are displayed as mean ± SEM of five mice per group. Statistics: unpaired two-tailed t-test.

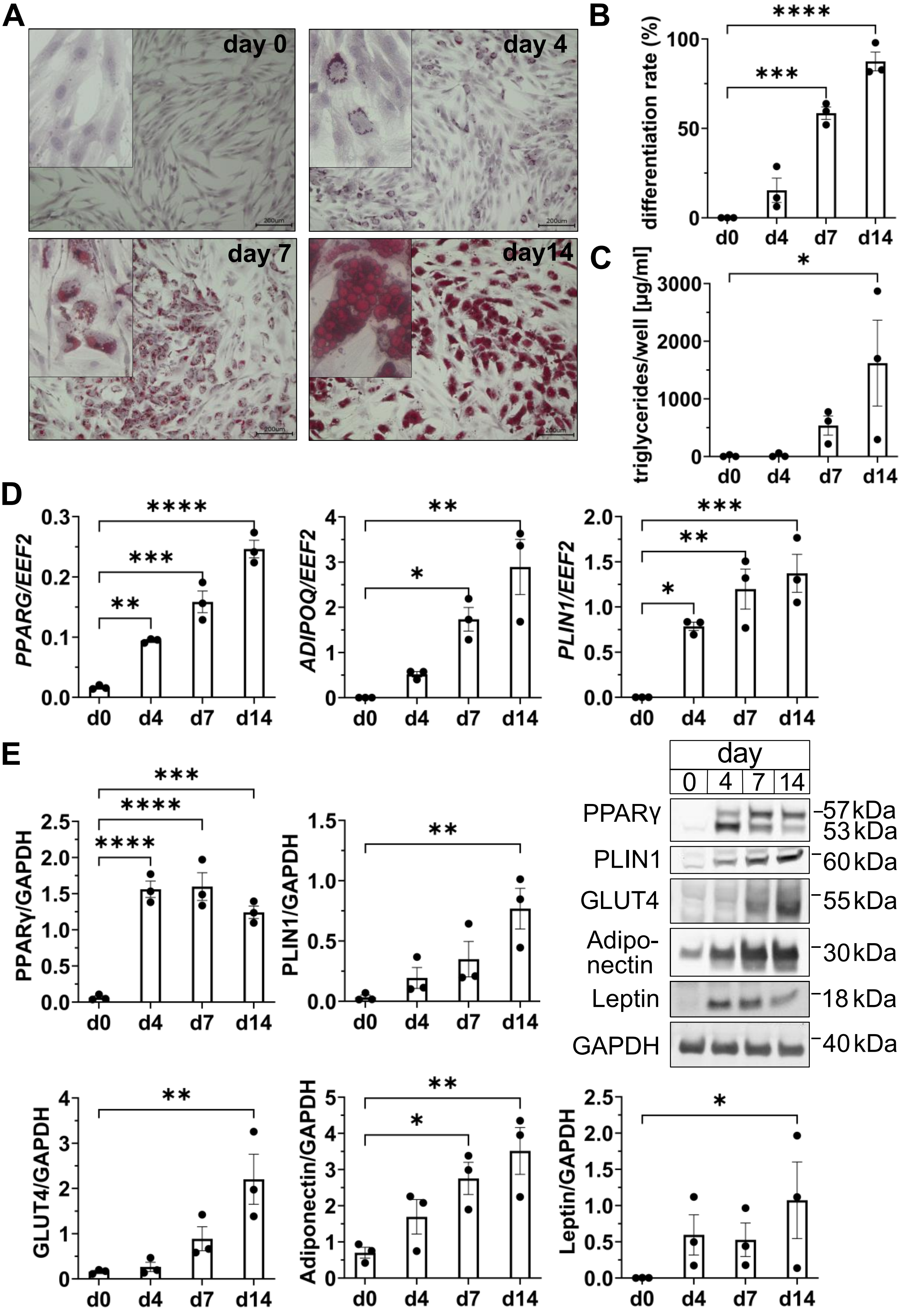
A
day 0
day 4
day 7
day14
B
differentiation rate (%)
d0
d4
d7
d14
C
triglycerides/well [µg/ml]
D
PPARG/EEF2
ADIPOQ/EEF2
PLIN1/EEF2
E
PPARγ/GAPDH
PLIN1/GAPDH
day
0
4
7
14
PPARγ
PLIN1
GLUT4
Adipo-
nectin
Leptin
GAPDH
57 kDa
53 kDa
60 kDa
55 kDa
30 kDa
18 kDa
40 kDa
GLUT4/GAPDH
Adiponectin/GAPDH
Leptin/GAPDH

Supplementary Figure S2. Adipogenesis of SGBS cells. SGBS cells were differentiated and analyzed on day 0, 4, 7 and 14. **(A)** Lipid droplets were stained with Oil Red O. Nuclei and cell bodies were stained with hematoxylin. Microscopic pictures were taken at 20x (zoomed in) and 10x magnification. **(B)** Differentiation rate, denoted in % and **(C)** triglyceride content per well of a 12-well-plate in µg/ml of SGBS cells during adipogenesis. **(D)** mRNA levels of PPARG, ADIPOQ, PLIN1 were measured by RT-qPCR in relation to EEF2 as reference gene and values were determined by using the ΔCt-method. **(E)** Protein expression of PPARγ, PLIN1, Leptin, Adiponectin, GLUT4 during the adipogenic differentiation process. One representative Western Blot out of three and densitometric analyses are shown using GAPDH as loading control. Statistics: *$p < 0.05$; **$p < 0.01$; p*** < 0.001; ****$p < 0.0001$; one-way ANOVA with Dunnett correction.

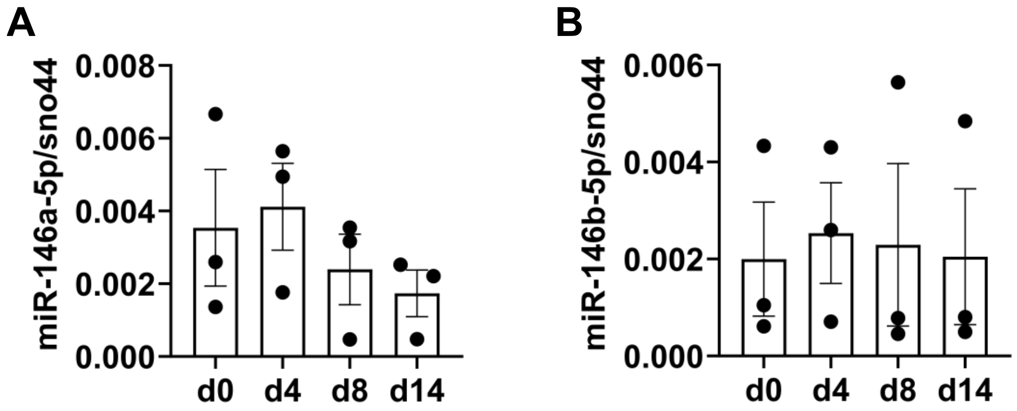

Supplementary Figure S3. Baseline expression of miR-146a/b-5p remains largely stable during adipogenesis. SGBS cells were differentiated and analyzed on day 0, 4, 7 and 14. **(A)** Baseline expression of miR-146a-5p. **(B)** Baseline expression of miR-146b-5p. Values are presented as mean ± SEM of three independent experiments. Statistics: one-way ANOVA with Tukey correction, ns.

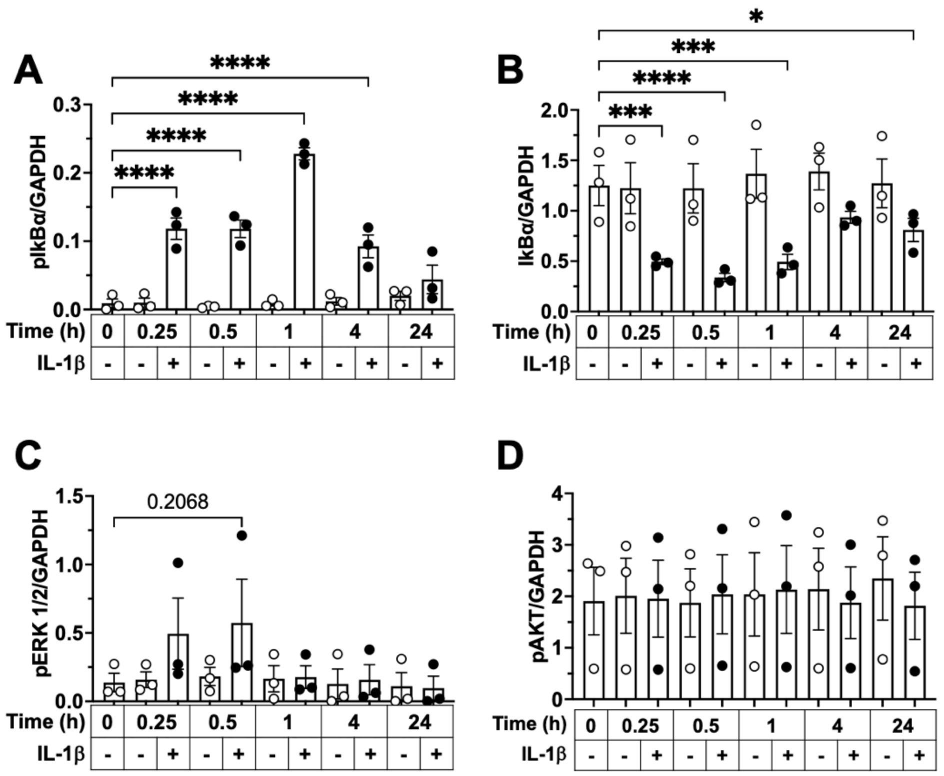

Supplementary Figure S4. Densitometric analysis of Figure 3A. SGBS adipocytes were treated with IL-1β or the corresponding vehicle control. Protein was extracted 0, 0.25, 0.5, 1, 4 and 24 hours post-stimulation. Protein expression of pIκBα **(A)**, IκBα **(B)**, pERK1/2 **(C)** and pAKT **(D)** were assessed by Western Blot and analyzed by densitometry in relation to GAPDH as loading control. The results are displayed as mean ± SEM of three independent experiments. Statistics: one-way ANOVA with Dunnett correction, *p < 0.05, p*** < 0.001, ****p < 0.0001.

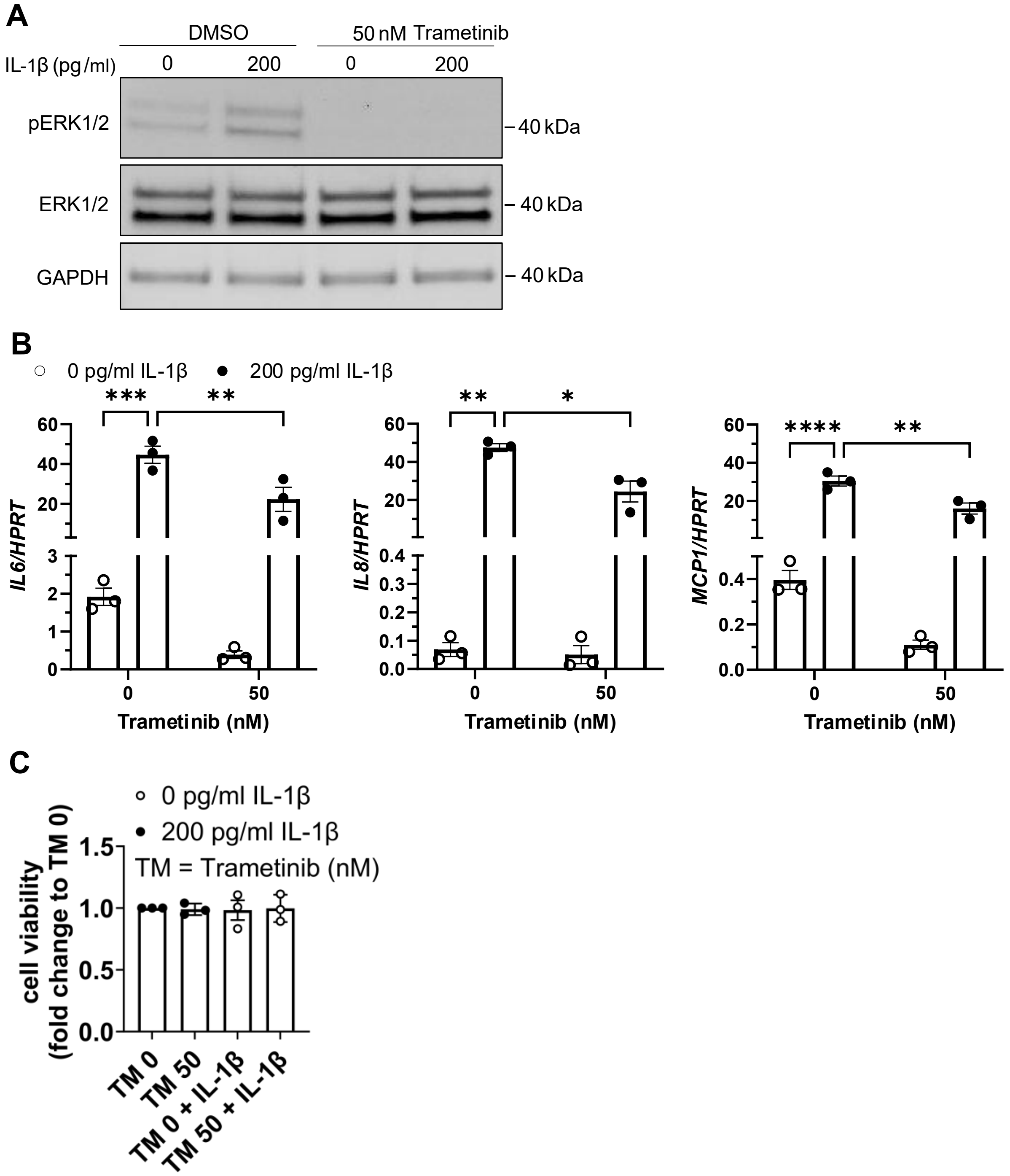

Supplementary Figure S5. MEK/ERK signaling contributes to inflammation induced by IL-1β in human adipocytes. SGBS adipocytes were incubated with DMSO (0 nM) or 50 nM trametinib (TM) for 30 minutes before stimulation with 200 pg/ml IL-1β or control for 4 h. **(A)** Protein expression of pERK1/2 and ERK1/2 is shown, GAPDH serves as loading control. One representative Western Blot out of four independent experiments is shown. **(B)** mRNA levels of *IL6*, *IL8*, and *MCP1* in relation to *HPRT* are shown. Values are determined by using the ΔCt-method. **(C)** SGBS adipocytes were treated with TM or DMSO as control in presence or absence of IL-1β (200 pg/ml) as indicated for 24 hours. Cell viability was measured by CellTiterGlo (CTG) assay. Values are presented as mean ± SEM of three independent experiments. Statistics: *p < 0.05; **p < 0.01; two-way ANOVA **(B)** and one-way ANOVA **(C**; ns**)** with Tukey correction.

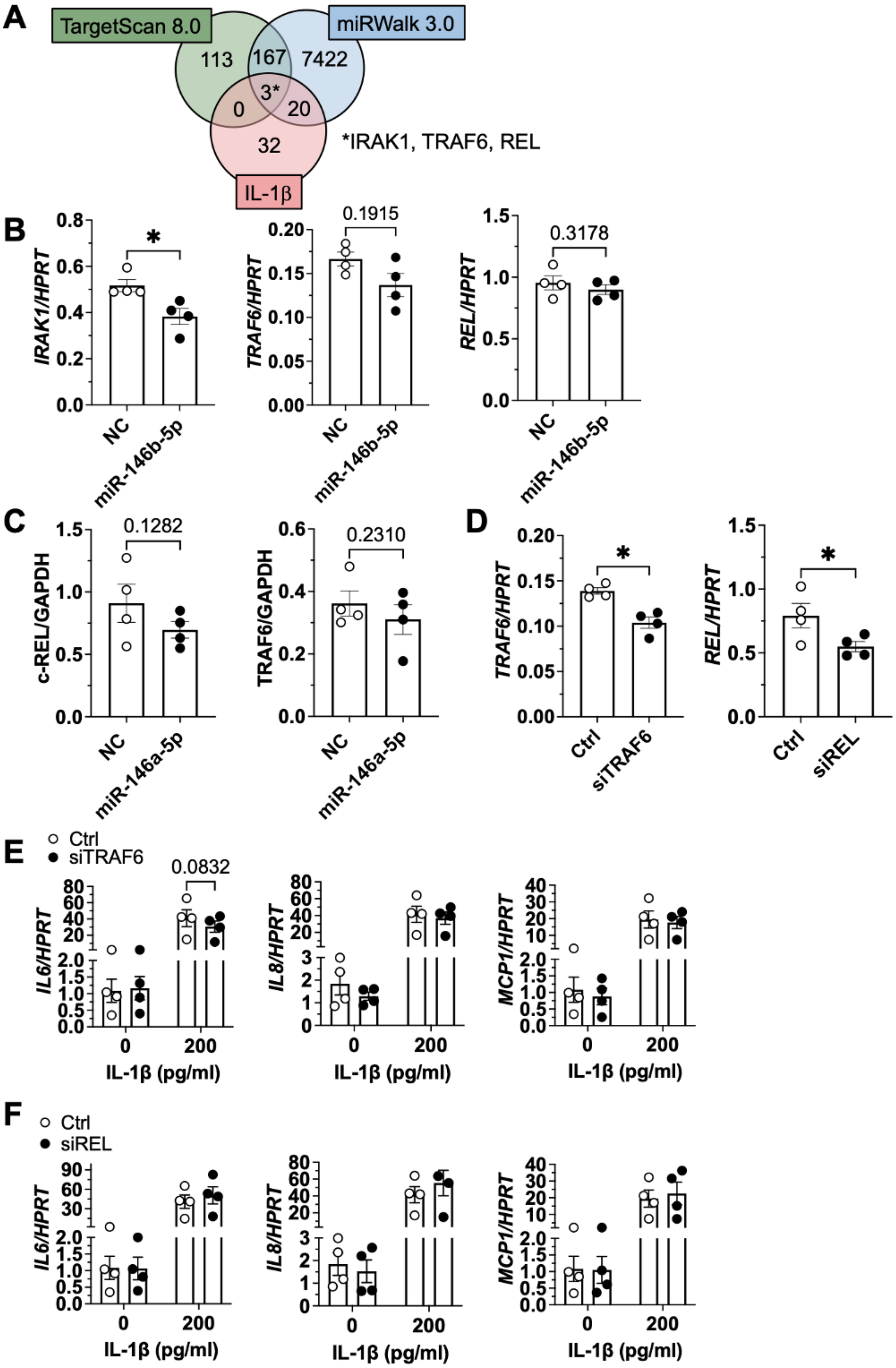
A
TargetScan 8.0
miRWalk 3.0
113
167
7422
3*
0
20
32
IL-1β
*IRAK1, TRAF6, REL
B
IRAK1/HPRT
*
NC
miR-146b-5p
TRAF6/HPRT
0.1915
REL/HPRT
0.3178
C
c-REL/GAPDH
0.1282
NC
miR-146a-5p
TRAF6/GAPDH
0.2310
D
TRAF6/HPRT
REL/HPRT
Ctrl
siTRAF6
siREL
E
Ctrl
siTRAF6
0.0832
IL6/HPRT
IL8/HPRT
MCP1/HPRT
IL-1β (pg/ml)
F
Ctrl
siREL

Supplementary Figure S6. Related to Figure 5. (A) Venn diagram representing the intersection of two *in silico* target gene predictions (TargetScan 8.0 and miRWalk 3.0) for miR-146b-5p and genes of the IL-1β signaling pathway (WikiPathway 195) resulting in an overlap of three genes, i.e. IRAK1, TRAF6, REL. **(B)** SGBS adipocytes were transfected with 50 nM miR-146b-5p mimic or a non-targeting control (NC). Total RNA was isolated 7 days post-transfection. *IRAK1*, *TRAF6* and *REL* mRNA levels were assessed by RT-qPCR using the ΔCt-method with *HPRT* as reference gene. The results are displayed as mean ± SEM of four independent experiments performed in triplicates. **(C)** SGBS adipocytes were transfected with 50 nM miR-146a-5p mimic or a non-targeting control (NC) (see Figure 5C). Protein was extracted 7 days post-transfection. Densitometric analysis of protein expression of TRAF6 and c-REL is displayed as mean ± SEM of four independent experiments performed in duplicates. **(D)** SGBS adipocytes were transfected with 20 nM TRAF6 or REL siRNA or control (Ctrl). mRNA levels were assessed by RT-qPCR using the ΔCt-method with *HPRT* as reference gene 72 h post-transfection. The results are displayed as mean ± SEM of four independent experiments performed in triplicates. **(E** and **F)** siRNA-transfected SGBS adipocytes were stimulated with IL-1β (200 pg/ml) or the corresponding vehicle control 72 h post-transfection. Total RNA was isolated 4 h after stimulation. *IL6, IL8, and MCP1* mRNA expression was analyzed. Statistics: paired two-tailed t-test **(B, C** and **D)**, two-way ANOVA with Šídák correction **(E** and **F)**.

Gene	Forward primer	Reverse primer
ADIPOQ	GGCCGTGATGGCAGAGAT	CCTTCAGCCCCGGGTACT
EEF2	GAGACACGCTTCACTGATACCCGG AAGGA	GTAGAAGAGGGAGATGGCAGTTGACTT GATGG
HPRT	GAGATGGGAGGCCATCACATTGTA GCCCTC	CTCCACCAATTACTTTTATGTCCCCTGTT GACTGGTC
IL6	TACCCCCAGGAGAAGATTCC	TTTTCTGCCAGTGCCTCTTT
IL8	TGCCAAGGAGTGCTAAAGAACTTA GATGTCAG	AGCTTTACAATAATTTCTGTGTTGGCGC AGTG
IRAK1	GGAGACATCAAGAGTTCCAACGTC CTTCTG	GTCTTTCAGATACTTGGTCCTGGCACCG T
MCP1	TCCCAAAGAAGCTGTGATCTTCAAG ACC	AGTGAGTGTTCAAGTCTTCGGAGTTTGG
PLIN1	GAAGTTGAAGCTTGAGGAGCGAGG ATGG	GGCTTCCTTAGTGCTGGTGTAGGTCTTC TG
PPARG	GATCCAGTGGTTGCAGATTACAA	GAGGGAGTTGGAAGGCTCTTC
REL	CAACCGAACATACCCTTCTATCC	TCTGCTTCATAGTAGCCGTCT

Supplementary Table S1. Human primer sequences used for qRT-PCR.

Name	Mature miRNA Sequence	Gene Globe ID
hsa-miR-200c-3p miRCURY LNA	5'UAAUACUGCCGGGUAAUGAUGGA	YP00204482
hsa-miR-34a-5p miRCURY LNA	5'UGGCAGUGUCUUAGCUGGUUGU	YP00204486
hsa-let-7f-5p miRCURY LNA	5'UGAGGUAGUAGAUUGUAUAGUU	YP00204359
hsa-miR-150-5p miRCURY LNA	5'UCUCCCAACCCUUGUACCAGUG	YP00204660
hsa-miR-15a-3p miRCURY LNA	5'CAGGCCAUAUUGUGCUGCCUCA	YP00204435
hsa-miR-203a-3p miRCURY LNA	5'GUGAAAUGUUUAGGACCACUAG	YP00205914
hsa-let-7g-5p miRCURY LNA	5'UGAGGUAGUAGUUUGUACAGUU	YP00204565
hsa-miR-146a-5p miRCURY LNA	5'UGAGAACUGAAUUCCAUGGGUU	YP00204688
hsa-miR-146b-5p miRCURY LNA	5'UGAGAACUGAAUUCCAUAGGCU	YP00204553
mmu -miR-146b-5p miRCURY LNA	5'UGAGAACUGAAUUCCAUAGGCU	YP02119752
SNORD44 (hsa) miRCURY LNA	-	YP00203902
SNORD68 (mmu) miRCURY LNA	-	YP00203911

Supplementary Table S2. miRCURY LNA miRNA PCR Assays used as miRNA PCR primers.

FC PT+HS vs Sham	MicroRNAID	murine sequence	human sequence	conserved?
5.14	mmu-miR-200c-3p	uaauacugccggguaaugaugga	uaauacugccggguaaugaugga	yes
4.28	mmu-miR-34a-5p	uggcagugucuuagcugguugu	uggcagugucuuagcugguugu	yes
3.06	mmu-miR-466j	ugugugcaugugcauguguguaa	-	no
2.98	mmu-miR-5620-3p	acagucaucccccugccucac	-	no
2.85	mmu-miR-21a-5p	uagcuuaucagacugauguuga	-	no
2.74	mmu-let-7f-5p	ugagguaguagauuguauaguu	ugagguaguagauuguauaguu	yes
2.69	mmu-miR-150-5p	ucucccaacccuuguaccagug	ucucccaacccuuguaccagug	yes
2.61	mmu-miR-15a-3p	caggccauacugugcugccuca	caggccauauugugcugccuca	one base diff.
2.6	mmu-let-7k	ugagguaggagguugugug	-	no
2.5	mmu-miR-203-3p	gugaaauguuuaggaccacuag	gugaaauguuuaggaccacuag	yes (203a-3p)
2.26	mmu-miR-7653-5p	uaaggggcggacagacagacgg	-	no
2.24	mmu-miR-3104-5p	uagggggcaggagccggagcccucu	-	no
2.22	mmu-let-7g-5p	ugagguaguaguuuguacaguu	ugagguaguaguuuguacaguu	yes
2.17	mmu-miR-5099	uuagaucgauguggugcucc	-	no
2.07	mmu-miR-146b-5p	ugagaacugaauuccauaggcu	ugagaacugaauuccauaggcug	yes
2.05	mmu-miR-7005-5p	ccuggggaugggaggaccagca	-	no
2.02	mmu-miR-342-3p	ucucacacagaaaucgcacccgu	ucucacacagaaaucgcacccgu	yes
2.02	mmu-miR-665-5p	aggggccucugccucuauccaggauu	accaggaggcugaggccccu (hsa-mir-665)	unknown

Supplementary Table S3. MiRNA candidates that were differentially regulated in iWAT depots 4 h after polytrauma and hemorrhagic shock (PT+HS) compared to sham-treated mice with a p-value ≤ 0.05 and a fold change (FC) ≥ 2.0 for PT+HS versus sham are shown.
